# Socio-hydrological modelling using participatory System Dynamics modelling for enhancing urban flood resilience through Blue-Green Infrastructure

**DOI:** 10.1016/j.jhydrol.2024.131248

**Published:** 2024-06

**Authors:** Virginia Rosa Coletta, Alessandro Pagano, Nici Zimmermann, Michael Davies, Adrian Butler, Umberto Fratino, Raffaele Giordano, Irene Pluchinotta

**Affiliations:** a Department of Civil, Environmental, Land, Construction and Chemistry, Polytechnic University of Bari, Bari, Italy; b Water Research Institute - National Research Council, Bari, Italy; c Institute for Environmental Design and Engineering, The Bartlett Faculty of the Built Environment, University College London, London, United Kingdom; d Department of Civil and Environmental Engineering, Imperial College London, London, United Kingdom

**Keywords:** Socio-hydrological modelling, Urban flood resilience, Stakeholder engagement, Blue-Green Infrastructure, Thamesmead

## Abstract

Cities are complex systems characterised by interdependencies among infrastructural, economic, social, ecological, and human elements. Urban surface water flooding poses a significant challenge due to climate change, population growth, and ageing infrastructure, often resulting in substantial economic losses and social disruption. Traditional hydrological modelling approaches for flood risk management, while providing invaluable support in the analysis of hydrological dynamics of floods, lack an understanding of the complex interplay between hydrological and non-hydrological (i.e., social, environmental, economic) aspects in an urban system, hindering effective flood risk management strategies. In this context, socio-hydrological modelling methods offer a complementary perspective to traditional hydrological models by integrating hydrological and social processes, thereby enhancing the understanding of the complex interactions driving flood resilience.

The present work proposes a participatory socio-hydrological modelling approach based on System Dynamics (SD) to quantitatively analyse the interactions and feedback between flood risk and different aspects of the urban system. By combining scientific expertise with stakeholder knowledge, the modelling approach aims to provide decision-makers with a comprehensive understanding of flood dynamics and the effectiveness of resilience-building measures. Furthermore, the role of Blue-Green Infrastructure (BGI) in enhancing urban flood resilience, considering its interplay with grey infrastructure and interactions with various sub-systems, is explored.

The results reveal i) the contribution of SD quantitative modelling in supporting the analysis of interactions between flood risk reduction measures and different sub-systems thus offering decision-makers actionable insights into the multifaceted nature of flood risk and resilience; ii) the added value provided by the combination of scientific and stakeholder knowledge in tailoring the model to the case study, quantifying socio-hydrological modelling dynamics limitedly explored in the scientific literature and supporting the selection of measures for increasing flood resilience; iii) the ability of BGI to provide not only hydrological benefits (mainly about the reduction of surface runoff) but also multiple social and environmental benefits (i.e., the co-benefits), especially when coupled with well-functioning grey infrastructure. Reference is made to one of the case studies of the CUSSH and CAMELLIA projects, namely Thamesmead (London, United Kingdom), a formerly inhospitable marshland currently undergoing a process of urban regeneration, with an increasing vulnerability to flooding.

## 1 Introduction

### 1.1 Setting the scene

Cities are complex systems that encompass infrastructural, economic, social, ecological, and human interdependence (Gao et al., 2022). These systems are characterised by dynamic and uncertain evolution due to the ever-changing relationships among their various components (Mannucci et al., 2022). Consequently, altering one element can have unforeseen effects on the entire system (Disse et al., 2020). Among the multitude of challenges that cities face, urban surface water flooding emerges as a frequent phenomenon. It occurs when intense/frequent precipitation cannot seep into the ground or be drained through natural or artificial systems (Evans et al., 2004) and usually leads to significant and prolonged economic losses due to damage to property, infrastructure, services, and human activities (Bosher, 2014). Societies and hydrological systems interact and influence each other in a co-evolutionary manner (Sivapalan and Blöschl, 2015). On one hand, the combined effects of climatic and socio-economic changes, such as increased frequency and intensity of extreme events, population growth, and expansion of impermeable surfaces, are raising the risk of urban flooding and its consequences (Friedman, 2008; Keesstra et al., 2018). On the other hand, flood episodes are affecting the evolutionary dynamics of urban systems (Walker et al., 2013). Limited understanding of this complex and long-term interplay between flood risk and urban dynamics can hinder the effectiveness of strategies for flood risk management (Kwakkel et al., 2010; Barendrecht et al., 2017). Therefore, decision/policy-makers cannot ignore these aspects and should move towards a holistic approach to flood risk management, i.e., capable of combining social and hydrological systems, while revealing complex interactions across scales, sectors, and groups (Di Baldassarre et al., 2015). Literature has shown that traditional hydrological modelling approaches for flood risk management in urban areas have limitations. Firstly, they fail to combine different sub-systems such as hydrological, social, economic, and environmental (Wamsler et al., 2013). Secondly, they overlook the existence of bidirectional feedbacks between flood and urban dynamics, such as built environment evolution, population growth and distribution, and infrastructure development (Ciullo et al., 2017). Focusing mainly on short- to medium-term physical (hydrological) aspects, traditional hydrological modelling approaches cannot consider the impacts of urban dynamics on flood risk management. In fact, assessing the effect of these dynamics requires a longer-term model simulation time scale (Di Baldassarre et al., 2019). This oversight has significant implications, as it neglects to recognize how urban changes influence the consequences of extreme flood events (and vice versa), as well as the selection of appropriate mitigation measures to reduce flooding impacts (Riddell et al., 2019; Genova and Wei, 2023). To illustrate, when a flood occurs, it may result in population displacement or loss of life. Traditional hydrological modelling approaches typically do not take into consideration the subsequent alterations in population density and distribution. These changes may, in turn, significantly influence the consequences of extreme flood events and the selection of effective flood mitigation measures. This perspective emphasizes the need to go beyond merely reacting to flood events and instead adopt a holistic approach that considers the dynamic and co-evolving nature of both urban areas and flood scenarios.

In this context, socio-hydrological modelling methods (see Blair and Buytaert, 2016 for further details) offer a complementary perspective to traditional hydrological modelling approaches for flood risk management. Although socio-hydrological modelling methods usually do not simulate hydrological components in detail, they have the potential to combine social and hydrological systems, providing a more accurate representation of long-term real-world dynamics (such as the urban dynamics) (Sivapalan et al., 2012). The fundamental assumption on which socio-hydrological modelling methods are based is that the dynamics of water-related issues, such as flooding, cannot be fully understood or effectively managed by studying either hydrologic or social systems in isolation (Di Baldassarre et al., 2013). Rather, the interconnections and feedbacks between different aspects of complex systems must be explicitly considered (McMillan et al., 2016). Based on that, socio-hydrological modelling methods provide an integrated framework to analyse the interdependencies and bidirectional feedbacks between flood and urban dynamics allowing for a more holistic understanding of complex systems (Viglione et al., 2014; Zwarteveen and Boelens, 2014; Barreteau et al., 2017). This means that these modelling approaches focus on the entire system (and not just on the phenomena) and embrace diverse perspectives (socio-cultural, economic, institutional, etc.), consequently supporting the identification of effective adaptive strategies that enhance system resilience (see e.g., Srinivasan et al., 2017; Jaramillo et al., 2018; Albertini et al., 2020). A shift from the paradigm of risk management to that of resilience management is therefore addressed by socio-hydrological modelling methods (Penny and Goddard, 2018). While risk management traditionally focuses on minimising the occurrence and impacts of specific hazards (Linkov et al., 2014), resilience management emphasizes the capacity of a system to absorb disturbances and adapt to change (Pahl-Wostl et al., 2007; Mao et al., 2017; Pagano et al., 2017; Cea and Costabile, 2022). Hence, this shift acknowledges that hazards and risks are inherent in natural and social systems, and it seeks to enhance the overall resilience of these systems. To summarise, socio-hydrological modelling methods are not proposed to replace traditional hydrological modelling approaches for flood risk management, which provide invaluable support in the analysis of hydrological dynamics of floods; rather, they are useful to understand the interactions between the phenomenon under investigation and the social dynamics within a complex system, while losing refinement in terms of the physical analysis of the hydrological phenomenon. Socio-hydrological modelling methods are therefore suggested to the hydrological community to complement the information obtained by traditional hydrological modelling approaches. In particular, the outputs of traditional hydrological models can be simplified and used as inputs in socio-hydrological models, combining them with social components and processes. Despite the potential of socio-hydrological modelling methods, the existing literature often overlooks the importance of stakeholder engagement throughout the different modelling stages. In fact, understanding the complex interactions of socio-hydrological systems requires knowledge combination (Sanders et al., 2020). This involves blending scientific data with expert and stakeholder insights (Voinov and Bousquet, 2010). Participatory modelling techniques facilitate this combination, aiding stakeholders in building conceptual models (Jordan et al., 2018) and grasping cross-sectoral connections (Moallemi et al., 2021). Stakeholder viewpoints can provide an invaluable lens through which adaptive strategies within socio-hydrological modelling can be selected, refined and optimized (Loucks, 2015). Firstly, stakeholder involvement brings different local knowledge into the assessment of the system dynamics supporting the identification of critical system components, feedback loops, and vulnerabilities that may be overlooked in non-participatory modelling approaches (Pluchinotta et al., 2021a). This improves the accuracy and relevance of system models, leading to more effective resilience-building strategies (Inam et al., 2017a,2017b,2017c). Secondly, stakeholders possess valuable knowledge about their social, cultural, and economic contexts, as well as their historical experiences with flood hazards (Perrone et al., 2020). Their involvement allows for the identification of locally appropriate and culturally sensitive adaptation measures that can effectively reduce vulnerability to flooding and enhance resilience (Mehryar and Surminski, 2022). Thirdly, by including stakeholders from various sectors and levels of governance, socio-hydrological modelling approaches foster cooperation, trust, and shared ownership of decision-making processes (Barreteau et al., 2017; Pluchinotta et al., 2018). This enhances the adaptive capacity of the system and facilitates the implementation of effective resilience-building measures (Pahl-Wostl, 2009). Despite these considerations, the level of stakeholder involvement in socio-hydrological modelling is still limited (Scaini et al., 2021), mainly due to skills, but also time and funding constraints, the lack of trust that decision/policy-makers have in participatory approaches, and the difficulty of combining different types of knowledge into a single model (Löschner et al., 2016).

In recent years, disasters triggered by flooding have demonstrated the need for management measures to minimize their impacts on the built environment, communities, and the economy. Combining Blue-Green Infrastructure (BGI) (i.e., measures that work with natural processes, deliver co-benefits, and “make space for water” such as wetlands, swales, and trees) with existing grey systems (i.e., hard, conventional, or engineering solutions such as dams and levees) is becoming a valid option (Fenner, 2017). BGI not only enhances the capacity of urban systems to adapt to urban flooding but also provides environmental, social, and economic co-benefits (Green et al., 2021; O’Donnell and Thorne, 2020b; O’Keeffe et al., 2022). In this context, stakeholder involvement is essential for identifying and maximizing the co-benefits associated with BGI implementation (Kabisch et al., 2017; Miller and Montalto, 2019; Giordano et al., 2020; Coletta et al., 2021). In fact, stakeholders provide valuable insights into the environmental, social, and economic challenges and opportunities in a given area, ensuring that the infrastructure is designed and managed to address those needs (Wickenberg et al., 2021). However, the lack of both technical support and decision-making tools for the evaluation of co-benefits represents a barrier in this regard (Alves, 2020). In addition, public acceptance issues and uncertainty about BGI’s long-term performance and costs constitute barriers to their implementation (IPCC, 2012; Wihlborg et al., 2019).

### 1.2 Objectives and research questions

Based on the above considerations, the objective of the present work is to develop a quantitative socio-hydrological modelling approach including stakeholder participation, capable of showing decision-makers i) a holistic view of the complex and dynamic behaviour of the flood-prone urban system, including (but not limited to) both hydrological and social aspects, processes and interactions; ii) the importance of combining scientific and stakeholder knowledge in both quantifying system relationships and selecting management measures that enhance urban flood resilience; iii) the benefits, co-benefits and impacts related to BGI implementation in urban areas, particularly focusing on its interplay with grey infrastructure and interactions with different sub-systems (i.e., hydrological, environmental, social, economic) and on their role in enhancing urban flood resilience. A participatory socio-hydrological modelling approach based on System Dynamics (SD) principles is implemented. It involves both qualitative and quantitative modelling phases, where scientific and stakeholder inputs are pivotal. While this work primarily focuses on the quantitative modelling phase, the qualitative phase is extensively discussed in Coletta et al. (2024). As underlined by the SD literature and practice, qualitative and quantitative modelling have different objectives and provide specific contributions (Sterman, 2000). Specifically, the SD qualitative modelling process described in Coletta et al. (2024), proposes the use of Causal Loop Diagrams (CLDs) for supporting the conceptual analysis of the complex interactions between flood risk assessment, risk reduction and urban dynamics, supporting preliminary qualitative assumptions about system dynamics and facilitating the discussion among stakeholders about flood risk management policies. Although the CLD guarantees an improved understanding of the system and supports the identification of potential points of intervention, it does not support the selection of effective adaptive strategies (Zomorodian et al., 2018a; Murphy, 2022). Due to this shortcoming, quantitative simulation models are indispensable and complementary. Building on the conceptual analysis and the qualitative system structure provided by the CLD, the SD model described in the present manuscript proposes a fully quantitative modelling approach and related simulation model, that helps to test different scenarios and thus identify effective flood management strategies, for instance to assess the interplay between the co-benefits and flood risk reduction of both BGI and grey infrastructure solutions. Insights from the qualitative modelling phase are used to refine quantitative model parameters and initial conditions, particularly in cases of data scarcity, thereby enhancing the accuracy and reliability of simulation results (Pluchinotta et al., 2024). Additionally, qualitative modelling insights contribute to better model validation.

Compared to traditional hydrological modelling approaches for flood risk management which mainly focus on hydrological (physical) processes, quantitative SD modelling applied to socio-hydrology supports the analysis of the interactions between physical (e.g., rainfall, surface runoff, etc.) and non-physical (e.g., citizen well-being, citizen preparedness to a flood event, etc.) aspects and between different sub-systems (e.g., hydrological, social, economic, etc.), and their combined effects (consequently highlighting feedback loops, potential trade-offs and unintended consequences) (Simonovic, 2009; de Vito et al., 2019). Unlike hydrological modelling approaches that usually focus on short/medium-term simulations of purely physical processes, quantitative SD modelling uses dynamic simulation to model how the entire system would behave under different assumptions over time, accounting for feedback loops and delays (Sterman, 2000). This is crucial e.g., for understanding the long-term consequences of different urban development dynamics or the impacts of specific flood risk management strategies (Ahmad and Simonovic, 2015; Karimlou et al., 2020). A key aspect that makes quantitative SD modelling useful in socio-hydrological contexts is its ability to effectively combine the results of sectoral models (e.g. hydrological, social, economic, etc.) (Zomorodian et al., 2018a). This means that the outputs of sectoral models can be used, albeit with some assumptions and simplifications, as inputs of the SD model thus contributing to the development of a full picture of the system under investigation. Furthermore, due to its graphic nature, SD modelling facilitates effective communication and collaboration with/between stakeholders (Senge and Sterman, 1992; Sušnik et al., 2018).

Within this context, the present work adopts a holistic perspective to the concept of socio-hydrological resilience to flooding, including a quantitative analysis of multiple dynamic mechanisms influencing flooding at the urban scale.

The work aims to address the following research questions: i) to what extent can quantitative System Dynamics modelling support decision-makers with a holistic understanding of a complex socio-hydrological system (such as the urban system), while taking into account its interactions?; ii) to what extent can the combination of scientific and stakeholder knowledge within quantitative System Dynamics modelling increase the understanding of both flood risk and consequences in urban settings as well as support the selection of measures for increasing flood resilience?; iii) To what extent does the implementation of Blue-Green Infrastructure – and the interplay with grey infrastructure – enhance urban flood resilience through the interaction with different sub-systems (e.g., environmental, economic, social, hydrological)?

The modelling approach, although replicable in different study contexts, is applied to one of the case studies of the CUSSH (Complex Urban Systems for Sustainability and Health, https://projectcussh.org/) and CAMELLIA (Community Water Management for a Liveable London, https://www.camelliawater.org/) projects, namely Thamesmead (London, United Kingdom), known for its ongoing urban regeneration and vulnerability to different flooding sources.

## 2 Overview of System Dynamics modelling applied to socio-hydrology

System Dynamics (SD) is a computer-aided modelling approach enabling a comprehensive understanding of complex dynamics systems (such as urban systems) (Forrester, 1969). It achieves this by incorporating feedback loops among their different components as well as supporting the assessment of the potential impacts of system disturbances (Meadows et al., 1972; Sterman, 2000). An important aspect is that SD modelling is applicable in situations where the physical basis of a relationship is uncertain or challenging to depict. This is because correlative relationships can serve as a basis for modelling in such situations (Blair and Buytaert, 2015). For these reasons, SD modelling is particularly suitable for analysing coupled socio-hydrological systems (Perera and Nakamura, 2023). Over the years, it has gained popularity in water research (see e.g., Zarghami et al., 2018; Phan et al., 2021 for literature reviews on this topic), with numerous studies applying it to investigate the dynamic behaviour of water systems. For example, Di Baldassarre et al. (2013) and Viglione et al. (2014) used SD modelling for conceptualising the interactions and feedback loops in settled floodplains; Dhungel and Fiedler (2016) analysed the implications in watershed management; Pluchinotta et al. (2018) developed an SD model for supporting decision-makers in irrigation water management, while Pagano et al. (2019) built a participatory SD model capable to quantitatively assess the effectiveness of Nature-Based Solutions to deal with flood risks and producing a multiplicity of co-benefits.

The strength of the SD modelling approach lies in its well-established and adaptable framework. This makes it suitable for various research fields and contexts. Usually, this modelling technique begins with system conceptualization, which involves applying System Thinking principles and constructing a Causal Loop Diagram (CLD) (Mirchi et al., 2012). The CLD serves as a qualitative model of the system feedback structure, allowing for the generation of hypotheses about the system dynamics and policy implementation (Coletta et al., 2021). Although qualitative conceptual models (such as CLDs) are essential for understanding complex systems (Richmond, 1994), they may be insufficient for identifying and testing adaptive strategies and supporting their implementation. SD quantitative models (such as Stock and Flow models) can describe system behaviour beyond qualitative diagramming as they are simulation models that use mathematical equations to represent complex systems and their dynamics over time (Forrester, 1961). Mathematical computations, typically in the form of differential equations, are applied to capture the interactions between different elements in the model (Sterman, 2000). Stock and Flow (SF) models are thus considered an evolution of CLDs (Teegavarapu and Simonovic, 2014). As defined by Sterman (2000), stocks are variables that accumulate or deplete over time, while flows represent how stocks change over time. Auxiliary variables can be used to describe processes within the model.

In recent years, researchers have increasingly explored the potential of SD modelling approaches for socio-hydrological modelling, mainly related to SD modelling ability to: i) combine the results of sectoral models (e.g., hydrological, social, economic, etc.) as well as scientific and stakeholder knowledge (Zomorodian et al., 2018b); ii) provide insights into the evolution of complex systems, considering both quantitative/physical and qualitative/non-physical aspects (Simonovic, 2009); iii) analyse the interactions and external influences on the system (Mirchi et al., 2012); iv) explore multiple long-term scenarios (Sterman, 2000). Several research gaps have also been highlighted: i) only a few works have combined scientific and stakeholder knowledge (Karimlou et al., 2020); ii) SD modelling is often limited to one sub-system (e.g., the hydrological), or two sub-systems (e.g., hydrological-social and hydrological-economic) (Davies and Simonovic, 2011); iii) validation has been limitedly performed, especially when social, economic, and political sub-systems are included (Blair and Buytaert, 2016). The present work builds on previous research, combining participatory methods with SD modelling techniques. The purpose is to combine different sources of knowledge into the socio-hydrological modelling and jointly analyse multiple sectors (i.e., hydrological, social, economic, and environmental), ultimately identifying effective measures to enhance urban flood resilience, including BGI.

## 3 Modelling approach

This section provides an overview of the proposed participatory modelling approach (Section 3.1). For the sake of brevity, it is presented in detail through its application to an interesting case study (Section 3.3), namely Thamesmead (London, United Kingdom).

### 3.1 Outline of the proposed participatory modelling approach

The proposed modelling approach (Table 1) is based on a multi-step modelling process of knowledge gathering (from both literature and experts) using SD modelling principles.

This modelling process is not entirely groundbreaking in the SD field as it incorporates various well-established modelling steps familiar to SD experts. The novelty of the modelling process proposed in this paper lies in its application to a socio-hydrological context in a participatory manner. Nevertheless, it can be replicated in other contexts. The modelling framework should be interpreted as flexible enough to be customized to specific needs (also in terms of procedural order and structure) and implemented with a different level of detail in each context. Therefore, not all applications will need the inclusion of all modelling steps.

The modelling process includes both a qualitative and quantitative modelling phase in which scientific and stakeholder knowledge plays a central role. This paper mainly focuses on the application of the quantitative modelling phase, while the qualitative phase is described in detail in Coletta et al. (2024).

While some participatory modelling techniques, like the construction of Behavior Over Time (BOT) graphs with stakeholders (Step 4), are included in the qualitative modelling phase, the flexibility of the proposed modelling approach allows for the possibility of bypassing some steps, such as Steps 4 and 5 (i.e., developing BOT graphs and using them to enhance CLD narrative). Instead, stakeholders can be engaged in constructing BOT graphs directly within the quantitative modelling phase to aid in model building and validation.

After providing a brief description of the steps in the qualitative modelling phase, Section 3.3 focuses on the three steps of the quantitative modelling phase (represented in white, in Table 1) applying them to the Thamesmead case study, which is particularly intriguing due to its ongoing urban regeneration and vulnerability to different flooding sources.

### 3.2 The Thamesmead case study from the hydrological point of view

**Fig. 1:**
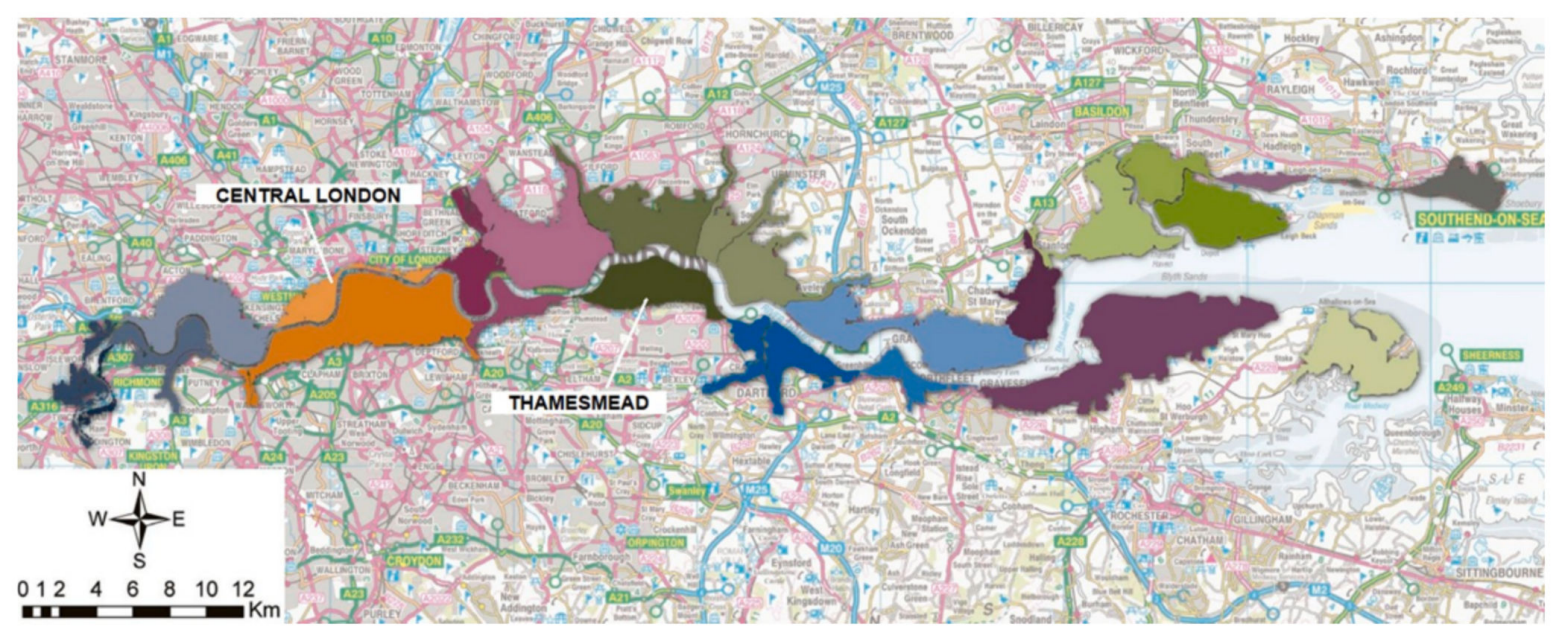
Study area overview in relation to central London, adapted from Environment Agency (2012). Coloured areas represent the eight Thames Estuary 2100 flood risk action zones.

Thamesmead (TM) is a former marshland in south-east London (United Kingdom), situated between the London Borough of Bexley and the Royal Borough of Greenwich. It is bordered by Woolwich to the southwest, Belvedere and Erith to the southeast, and the tidal River Thames to the north (Fig. 1) (Peabody, 2019).

The information in this Section was mainly gathered from the Thames Estuary 2100 Plan (Environment Environment Agency, 2012), the Living in the Landscape Framework (Peabody, 2021), the Charlton to Bexley Riverside Integrated Water Management Strategy (AECOM, 2017), Pluchinotta et al., (2021b), and from semi-structured interviews or personal correspondence with stakeholders (see Table 2 below for the list of the main stakeholders involved in the whole process). Initially intended for residential development due to its proximity to London, the full residential potential of the area was never realised (Markowitz, 2017). Recently, a regeneration plan has renewed interest in addressing flood risk. The plan aims to bring significant growth to the area over the next 20 years. The vision focuses on key aspects such as nature, connectivity, inclusion, safety, and resilience to the impacts of climate change. During other participatory modelling activities within the CUSSH (Complex Urban Systems for Sustainability and Health, https://projectcussh.org/) and CAMELLIA (Community Water Management for a Liveable London, https://www.camelliawater.org/) projects related to the definition of actions for increasing the quality of Built/Blue-Green environment in TM (see Pluchinotta et al., 2021b for further details), stakeholders emphasised the importance of building resilience to flooding to protect the local community and the built environment in TM. In fact, the area is vulnerable to four types of flooding: tidal, fluvial, pluvial, and groundwater. It is located in the tidal portion of the River Thames, experiencing the impacts of regular tides and potential water level increases from North Sea surges. It benefits from Thames Tidal Defences (a river wall and two sections of embankments), which protect against tidal flooding. The consequences of a breach would be significant, and considering the effects of ageing and climate change, the defences may need future improvements or replacement. The Wickham Valley watercourse is a main river that flows in the area and discharges into a surface water drainage network of lakes and canals, which is London’s largest Sustainable Urban Drainage System. Along with this system, a network of ditches and dykes (i.e., the Erith Marshes system) drains the area and flows into the River Thames. Both in the lakes and canals network and the Erith Marshes system the water levels are controlled by sluices and pumping stations.

TM is covered by the Crossness sewer network, which is primarily a combined sewer system. This means that both stormwater and foul (wastewater) flow through the same network of pipes. However, there are also areas with separated sewers in which distinct conduits are designated for wastewater and stormwater. The stormwater from separated sewers discharges into the lakes and canals. The study area consists primarily of reclaimed marshland and therefore has a high water table. As the base geology is relatively permeable, TM is considered to have the potential for groundwater flooding. Discussions with stakeholders revealed that flooding events in the area are mainly caused by the ageing and sediment build-up in both the stormwater system and the lakes and canals system, with additional contributions from groundwater and tidal river interactions. Stakeholders emphasised the importance of assessing the combined impact of these different flooding sources (i.e., pluvial, fluvial, tidal, groundwater) also with other aspects, such as environmental, economic, and social factors. Based on all these considerations, the integration of flood risk management into urban regeneration dynamics has been explored. Furthermore, the COVID-19 pandemic prompted the research team to adapt due to limitations in meetings, leading to virtual sessions involving a specific interest group. Due to these constraints, extensive stakeholder engagement was not possible for this model. Instead, a pertinent group of local technical stakeholders, mainly with a technical background (flood experts, representatives of relevant organizations or institutions as reported in Table 2) was identified, guaranteeing as much as possible the diversity in the background. Stakeholders were mainly asked to contribute to the development of parts of the model where the available information or data were scarce, providing expert knowledge. Specifically, during the first round of interviews described in Coletta et al. (2024), each interviewee who mentioned the issue of flooding was asked to suggest the involvement of another relevant stakeholder (in terms of role and expertise) to be invited in the following meetings (the snowballing approach described in Reed et al., 2009 was used). Considering the context and overall constraints described above, one of the aims of this modelling process was also to build a long-lasting engagement and relationship with/between stakeholders with an interest in flooding, to overcome their way of working in silos. Moreover, since the modelling focus, boundaries and objectives were defined with the stakeholders as part of the two research projects (CUSSH and CAMELLIA), the participatory modelling process presented in this paper could be defined as ‘expert-based’ (Barreteau et al., 2010). These restrictions related to the pandemic (in terms of number of participants and online participation) did not contradict the point on the importance of involving stakeholders in socio-hydrological modelling. Participatory modelling can be performed in many different forms which require a different stakeholder ‘sample size’. Although statistical analyses, performed e.g., through surveys or questionnaires, might require a relevant number of participants, participatory SD modelling can be performed with a relatively variable number of stakeholders/experts (Voinov and Bousquet, 2010) in the form of workshops or focus groups. However, it is recommended, especially in situations of data scarcity, to involve more stakeholders with different backgrounds.

### 3.3 Application of the modelling approach to the thamesmead case study

The modelling process included both a qualitative and quantitative modelling phase. Within the qualitative modelling phase (detailed in Coletta et al., 2024), Steps 1, 2 and 3 were about combining information from various sources (reports, existing models, etc.) and local stakeholder knowledge (collected via semi-structured interviews and workshops). The aim was to develop a qualitative conceptual model, namely a Causal Loop Diagram (CLD), that establishes connections between flooding and the key urban dynamics within the study area. CLD provided insight into the relationships between components inside (and outside) the system. In Step 4 stakeholders, during a workshop, drew Behaviour Over Time (BOT) graphs of key variables of the system under three different conditions (i.e., desired future, most likely future, and feared future) (see e.g., Elias, 2012 for further information on BOT graphs). The key variables were: ‘damage due to flooding,’ ‘quality of BG public spaces,’ ‘attractiveness of local area,’ and ‘residents’ health’. These variables were chosen for a twofold reason. First, they represent some of the objectives set by Peabody’s ambitious Plan for regeneration in TM. Second, the possibility of finding data that describe them over time is limited. Specific initial values and thresholds of the variables were also identified by participants. BOT graphs were then used in Step 5 to improve the CLD narrative (and more precisely the feedback loops) and formulate preliminary and realistic hypotheses on urban dynamics and flood risk management strategies.

The present paper focuses on the quantitative modelling phase, specifically the process from building the quantitative SD model, namely the Stock and Flow (SF) model, to identifying the most effective strategy for enhancing the resilience of the system to flooding. All the steps are detailed in the following sub-sections.

#### 3.3.1 Steps 1 and 2 – Construction and validation of the stock and flow model

Step 1 concerned the development of the SF model starting from the CLD built during the qualitative modelling phase. The model was created with the Vensim® software, a powerful simulation tool designed to enhance the performance of real systems (https://vensim.com/). The CLD variables and causal relationships were translated into SF model sets, including stocks, flows, auxiliary variables, and connectors as detailed in Section 2. Hypotheses on mathematical equations and parameters were formulated by combining multiple information sources (i. e., scientific/grey literature, reports, and databases) and stakeholder consultation. Specifically, stakeholder knowledge was acquired using email correspondence and BOT graphs from the qualitative modelling phase. BOT graphs related to the most likely evolution of the system were employed at this stage for quantifying the baseline values of key variables in the SF model for which there is a lack of data/information, or which are difficult to quantify. To handle the system complexity, the model was organised into interconnected thematic sub-models, representing key processes and elements of TM exposed to flood risk. Specifically, sub-models related to flood risk assessment, tangible damage evaluation, and co-benefits analysis were developed. Considering that the obtained information needs to be synthesised for planning and strategic purposes, some indices that aggregate representative variables of the behaviour of the system were constructed; these included the ‘flood hazard index,’ the ‘flood vulnerability index,’ the ‘flood risk index,’ and the ‘urban performance index’. The TM SF model ran over a time scale of 78 years (2023–2100), taking into account both the period covered by the regeneration plan (ending in 2050) and the future time horizon considered by the flood risk management plan. The combined effect of the different flooding sources in TM (i.e., fluvial, pluvial, tidal and groundwater) as well as of environmental, economic, and social factors was simulated. A daily time step was used as a compromise between that generally used for analysing urban drainage systems (i.e., sub-hourly/hourly) and that used for computing river and groundwater dynamics (monthly and/or yearly).

Step 2 concerned the validation of the SF model through stakeholder participation. Specific model inputs/outputs not covered in the BOT graphs drawn by stakeholders were validated through semi-structured interviews during an online workshop (approximate duration 1 h) (see Section 1 of the Supplementary Material for the workshop agenda). Stakeholders were asked about their agreement with assigned values for inputs and with the trends for outputs and were encouraged to provide justifications for their responses. Some model outputs covered in the BOT graphs were shown and discussed only in the case of deviation from stakeholder-built BOT graphs. Stakeholder suggestions were then implemented in the final SF model.

**Fig. 2:**
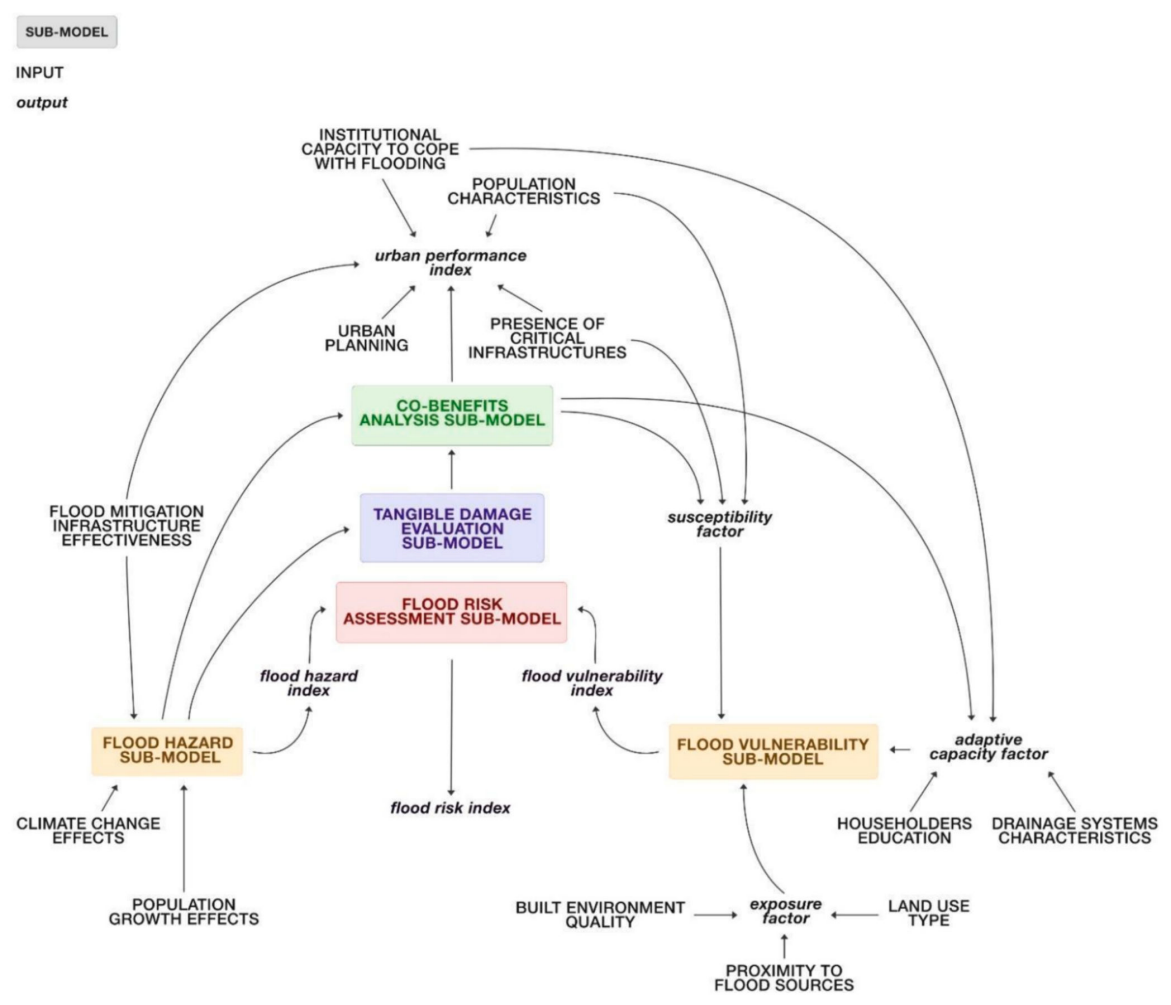
Interactions between the different SD sub-models proposed for the analysis of urban flood resilience. The sub-models are in coloured rectangles, while the main inputs/outputs of the sub-models are respectively in capital letters and italics.

A description of the SF model is provided below, organized based on the division into sub-models (Fig. 2). The list of variables, mathematical equations, data, initial values, and references behind the model are included in Section 2 of the Supplementary Material.

##### 3.3.1.1 Thamesmead ‘flood hazard’ sub-model description

**Fig. 3:**
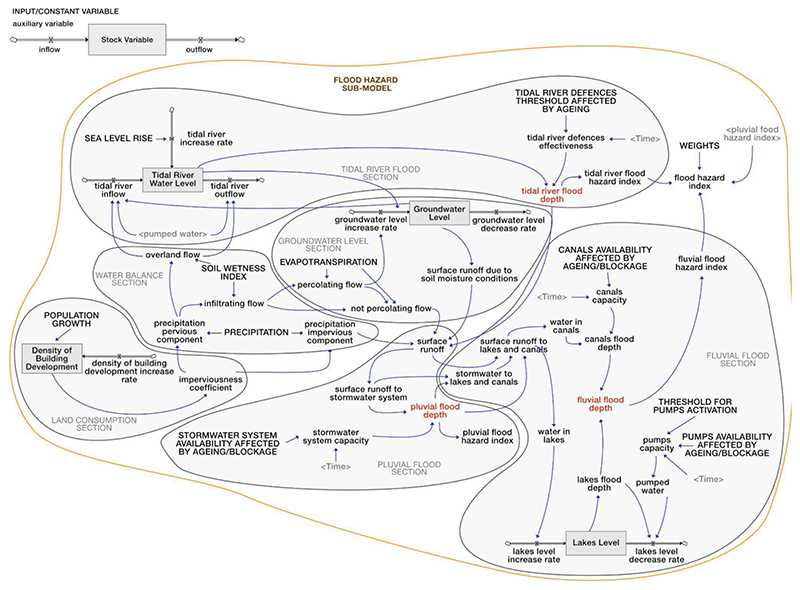
Thamesmead ‘Flood hazard’ sub-model. Sub-model parts are represented with grey circles. Variables in capital letters are the main inputs of the simulation, while variables in red are the main outputs in terms of simulated flooding mechanisms. (For interpretation of the references to colour in this figure legend, the reader is referred to the web version of this article.)

The ‘Flood hazard’ sub-model provided a simplified hazard assessment. This sub-model has been conceptually organized into six interdependent parts identified in Fig. 3 in grey circles, namely: ‘land consumption,’ ‘water balance,’ ‘groundwater level,’ ‘tidal river flood,’ ‘pluvial flood,’ and ‘fluvial flood’.

The variables in capital letters in Fig. 3 identify the input data for the simulation. These are hydrological variables affected by climate change (i.e., ‘precipitation,’ ‘evapotranspiration’ and ‘sea level rise’) as well as population dynamics and variables related to flood mitigation infrastructure effectiveness (e.g., ‘stormwater system availability affected by ageing/blockage,’ ‘tidal river defences threshold affected by ageing’). The UK Climate Projections (UKCP09) downscaled from the Met Office regional climate model were implemented in both precipitation and evapotranspiration timeseries. The highest changes in both the annual mean precipitation and potential evapotranspiration, considering a 90 % probability level under a high-emission future scenario, projected for the 2080 s relative to the baseline period of 1961–1990, were considered and distributed to the daily time scale (see Murphy et al., 2009; Thompson, 2012). To include the probabilistic component in the hazard assessment, the precipitation dataset was manually modified implementing several events with 2, 5, 10, 30, 50, 100 and 500-year future return periods. For this purpose, the 24-hour cumulated rainfall Intensity Duration Frequency curves for the City of London under changing climatic conditions were used (see Prodanovic and Simonovic, 2007). Future values for sea level rise in the Southeast of the UK were taken from Environment Environment Agency (2010).

The effect of population growth was also taken into account based on population projections (Askew, 2018) and expressed through changes in the ‘density of building development’, and consequently on soil imperviousness, over time. The effectiveness of flood mitigation infrastructure (i.e., the stormwater system, lakes and canals system, dykes system) was considered referring to infrastructure water storage capacity, which can be influenced by ageing and frequent sediment build-up. To this aim, future projections of systems clogging were developed based on past flooding episodes from this infrastructure. The spatial component of the pipe network was not explicitly considered, nor was the flow within the pipe network calculated. The effectiveness of the Thames Tidal Defences (i.e., a river wall and two sections of embankments) was also introduced as input in the model by considering the rate of height deterioration from past records (see the Thames Estuary, 2021).

The variables in red in Fig. 3 identify the main outputs in terms of simulated flooding mechanisms, i.e., ‘pluvial flood depth,’ ‘tidal river flood depth’ and ‘fluvial flood depth’. Since, according to stakeholders, flooding events occur in the area mainly with reference to the stormwater system and drainage systems (i.e., lakes and canals and dykes), groundwater flooding was not explicitly modelled. Instead, the ‘groundwater level’ was analysed based on data from the UK Environment Agency to understand both the amount of water contributing to ‘surface runoff’ (which feeds into both the stormwater and drainage systems) and the ‘tidal river level,’ modelled at high tide for simplicity. The amount of water flooding from the tidal river Thames was considered an additional contribution to the ‘surface runoff’.

To obtain a ‘flood hazard index’ summarising the sub-model information, each flood depth (i.e., ‘pluvial flood depth,’ ‘tidal river flood depth’ and ‘fluvial flood depth’) was associated with a hazard class between 1 (very low/low hazard) to 3 (high/very high hazard) adapting methods in Tingsanchali and Promping (2022). Weights were then assigned to the flood sources (the sum of which did not exceed 1), based on the capacity of individual flood sources to generate an impact on the system. A weighted average was then performed to obtain a global flood hazard index (FHI), based on both literature and stakeholders’ knowledge (elicited through semi-structured interviews and a workshop carried out during the qualitative modelling phase). The FHI, expressed in dimensionless terms, ranges from 1 (low index value) to 3 (high value). As mentioned above, all the inputs of the ‘Flood hazard’ sub-model were validated with the stakeholders during an online workshop (see Section 1 of the Supplementary Material for the workshop agenda). Stakeholders were asked for their agreement on the assigned values/classes/weights for each variable. Any disagreements prompted discussions and provided an opportunity for stakeholders to propose modifications, aiming to reach a consensus. For detailed information on the values/classes/weights assigned to each variable, refer to Section 2 of the Supplementary Material.

##### 3.3.1.2 Thamesmead ‘tangible damage evaluation’ and ‘co-benefits analysis’ sub-models description

**Fig. 4:**
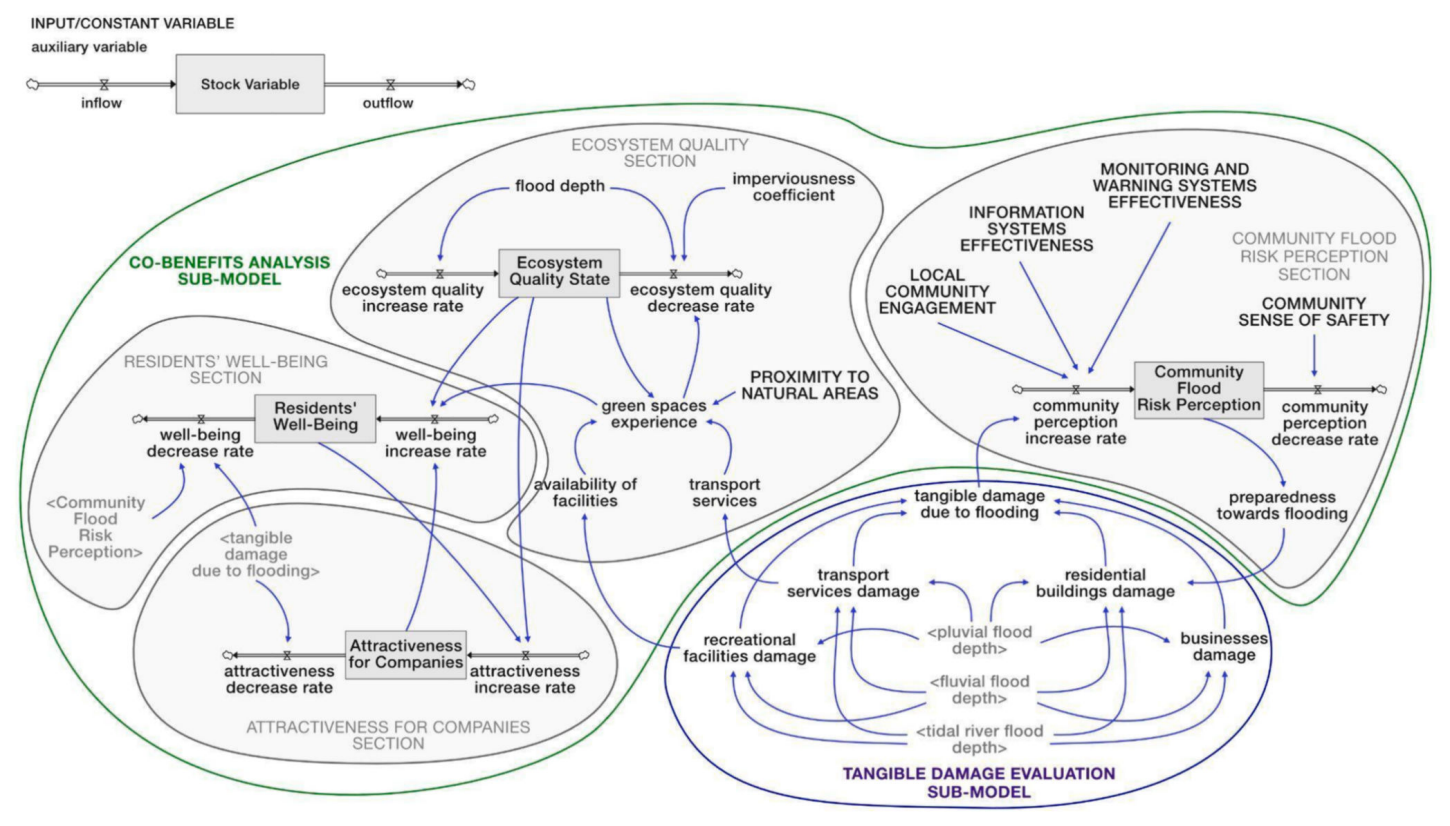
Thamesmead ‘Tangible damage evaluation’ and ‘Co-benefits analysis’ sub-models. The former is circled in blue, while the latter is in green. Sub-model parts are represented with grey circles. (For interpretation of the references to colour in this figure legend, the reader is referred to the web version of this article.)

The ‘Tangible damage evaluation’ sub-model (circled in blue in Fig. 4) was developed based on the flood depths calculated in the ‘Flood hazard’ sub-model. Depth (m) – damage (€/m2) curves, created by Penning Rowsell et al. (2010) for the UK, were applied to characterise the primary impacts of flooding on the built environment, including residential buildings, businesses, transport services, and recreational facilities. A global damage class for the built environment was developed to summarize the information from this sub-model and make it useful for future discussions with decision-makers. After calculating the damage for each component of the built environment, classes ranging from 1 (very low/low potential damage) to 3 (high/very high potential damage) were assigned using the methods outlined in Tingsanchali and Promping (2022). The global damage class for the built environment was then determined as the highest damage class among the various impacts, thus orienting the model in favour of safety.

Although evidence has demonstrated that co-benefits may be the main driver for the implementation of solutions, and mainly sustainable solutions (McVittie et al., 2018), few works have explicitly used a co-benefits analysis for the selection and design of BGI for flood mitigation (see e.g., Alves, 2020; Coletta et al., 2021). The ‘Co-benefits analysis’ sub-model (circled in green in Fig. 4) aimed to investigate the additional positive effects that planning and/or policy measures might have on social, environmental, and economic aspects of the urban system. The first step involved the identification of co-benefits for TM. These co-benefits were selected based on the objectives outlined in Peabody’s ambitious regeneration plan for the area, particularly focusing on creating an attractive neighborhood, enhancing the quality of BG public spaces, and improving residents’ well-being. Considering the difficulty of finding data or information in the literature to quantify the co-benefits, BOT graphs of key variables of the system (i.e., ‘quality of BG public spaces,’ ‘attractiveness of local area,’ and ‘residents’ health’), constructed by stakeholders during the qualitative modelling phase, were used to define the current value of each co-benefit. Once identified, the co-benefits were transformed into stocks as they are variables whose memory needs to be preserved over time in the system. As co-benefits are generally intangible variables, they were expressed in dimensionless terms, measured on a scale from 0 to 1 (or as a percentage), where 0 corresponds to the minimum level of the co-benefit, and 1 to the maximum level. The influence of some aspects from other sub-models on the co-benefits was also assessed through a literature review. For example, the impact of flood hazards on some population-related aspects, such as residents’ well-being and the attractiveness of the area, was explored. All parameters and connections within these sub-models were confirmed and validated through discussions with stakeholders during the online workshop.

##### 3.3.1.3 Thamesmead ‘flood vulnerability’ and ‘flood risk assessment’ sub-models description

**Fig. 5:**
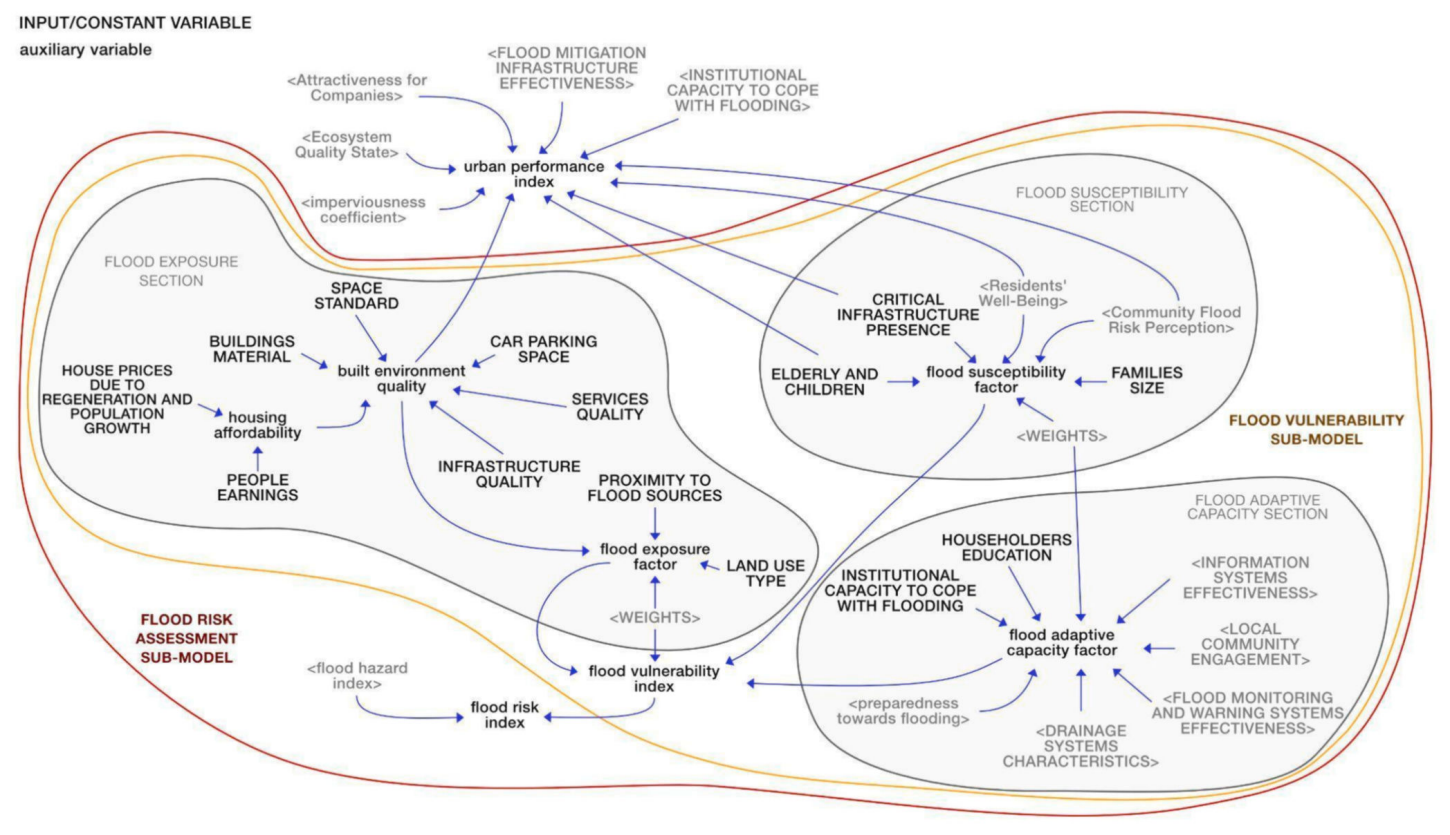
Thamesmead ‘Flood vulnerability’ and ‘Flood risk assessment’ sub-models. Sub-model parts are represented with grey circles.

The ‘Flood vulnerability’ sub-model (circled in orange in Fig. 5) provided a simplified vulnerability assessment, combining the exposure, susceptibility, and adaptive capacity in the face of the hazard (IPCC, 2012). Input variables (such as ‘people earnings,’ ‘families size,’ and ‘householders education’), represented in capital letters in Fig. 5, were initially defined based on existing literature and/or prior discussions with stakeholders (refer to Coletta et al., 2024).

To develop an aggregated index useful for future discussions with decision-makers, the variables were made comparable assigning each of them a class ranging from 1 (indicating very limited or non-existent condition/availability) to 3 (representing optimal condition/availability). Additionally, a weight was assigned to each variable (the sum of which did not exceed 1), reflecting its relative significance within its respective component (i.e., exposure, susceptibility, or adaptive capacity). The classes and weights were determined by modellers based on case study reports and/or prior discussions with stakeholders. Following the methodology of Kissi et al. (2015), Ntajal et al. (2016), Babanawo et al. (2022), and Tingsanchali and Promping (2022), the exposure factor (E), susceptibility factor (S), and adaptive capacity factor (AC) were determined by calculating the weighted average of the variables within each vulnerability component. To evaluate the overall flood vulnerability in TM, a global flood vulnerability index (FVI) was computed by combining the three factors (E, S, and AC) through the additive function w1E + w2S – w3*AC (w1, w2, and w3 represent the weights assigned to each factor, the sum of which did not exceed 1). The FVI, expressed in dimensionless terms, ranges from 1 (low index value) to 3 (high value).

Subsequently, the values of input variables, classes and weights were validated with the stakeholders during the online workshop. A simplified estimation of flood risk in the area was achieved by considering the interaction between hydrological, social, environmental and economic aspects (such as ‘flood depth’, ‘residents’ well-being’, ‘land use type’ and ‘housing affordability’). In particular, the ‘flood vulnerability index’ (FVI) was multiplied by the ‘flood hazard index’ (FHI) to derive a ‘flood risk index’ (FRI). The resulting FRI was categorized into three ranges: very low/low flood risk (class 1) for 1 < FRI ≤ 10, medium flood risk (class 2) for 10 < FRI ≤ 15, and high/very high flood risk (class 3) for 15 < FRI ≤ 25.

##### 3.3.1.4 Thamesmead resilience to flooding

To provide a comprehensive overview of the resilience of TM to flooding, an ‘urban performance index’ was calculated (see Fig. 5). Based on several studies (e.g., Cutter et al., 2010; Batica et al., 2013; Rockefeller, 2015; Figureido et al., 2018; Moghadas et al., 2019; Feofilovs et al., 2020; Marasco et al., 2022), the resilience was quantified distinguishing the characteristics of the system across five resilience dimensions (social, economic, institutional, infrastructural, and environmental). Social resilience referred to the capacity of the Thamesmead population groups to effectively respond in times of flooding. Economic resilience measured the vitality and resourcefulness of the local economy. Institutional resilience was connected with planning, preparedness initiatives and institutional capacity to cope with flooding. Infrastructural resilience related to physical assets that contribute to response and recovery capacity. The environmental dimension considered aspects of the urban environment that can increase or reduce flood risk. Variables of Thamesmead related to these dimensions of resilience (such as ‘elderly and children,’ ‘attractiveness for companies,’ ‘institutional capacity to cope with flooding,’ etc.) were selected based on the studies listed above and assigned a dimensionless class, ranging from 1 (indicating very low/low value) to 3 (representing very high/high value). To obtain a global urban performance index useful for planning/strategic purposes, weights were assigned to each variable and an aggregated average was performed. The classes and weights were initially given by the modellers based on case study reports and/or prior discussions with stakeholders and then validated with stakeholders during the online workshop.

#### 3.3.2 Step 3 – Future scenario building

Step 3 was centred on the identification of actions to increase the resilience of the urban system to flooding. The SF model developed during the previous steps was used with a twofold objective: i) assessing the long-term impacts of the baseline conditions; ii) performing a scenario analysis to understand the potential effect of the introduction of specific measures (including BGI) on TM flood resilience. A preliminary set of flood risk management scenarios was designed based on both Peabody’s regeneration plan for TM and previous discussions with stakeholders. These scenarios were then validated with stakeholders during an online workshop (approximate duration 1 h). The workshop agenda used for scenario building is included in Section 1 of the Supplementary Material. A facilitator presented the rationale of each scenario and stakeholders were asked whether they agreed with the behaviour over time of the representative variables of the system (i.e., ‘flood hazard index,’ ‘tangible damage due to flooding,’ ‘ecosystem quality state,’ ‘residents’ well-being,’ ‘community flood risk perception,’ ‘attractiveness for companies,’ ‘flood vulnerability index,’ ‘flood risk index,’ and ‘urban performance index’) in each scenario. Additional scenarios to be tested were also co-designed during an interactive discussion. A description of the scenarios is provided below (see Table 3), while full details on the variables changed in each scenario are included in Section 3 of the Supplementary Material.

To identify effective strategies for Thamesmead, the scenarios proposed in Table 3 were then combined and compared using the baseline condition as a reference. The combined scenarios are described in Table 4.

Lastly, sensitivity analysis was performed to identify the most influential variables affecting the resilience of the system, calculated in this work through the ‘urban performance index’. Identifying a significant shift in system behaviour due to a change in a parameter value can pinpoint a leverage point in the model, i.e., a parameter that can significantly affect the behaviour mode of the system (Breierova and Choudhari, 1996). The analysis involved varying most of the model parameters individually (+/- 50 % in separate runs) (Mateus and Franz, 2015). This analysis served a practical purpose by helping identify factors or processes that warrant continuous monitoring due to their potential impacts. Additionally, the analysis highlighted key elements that should be targeted to adapt and adjust strategies.

## 4 Results – Scenario analysis

### 4.1 Scenario comparison

**Fig. 6:**
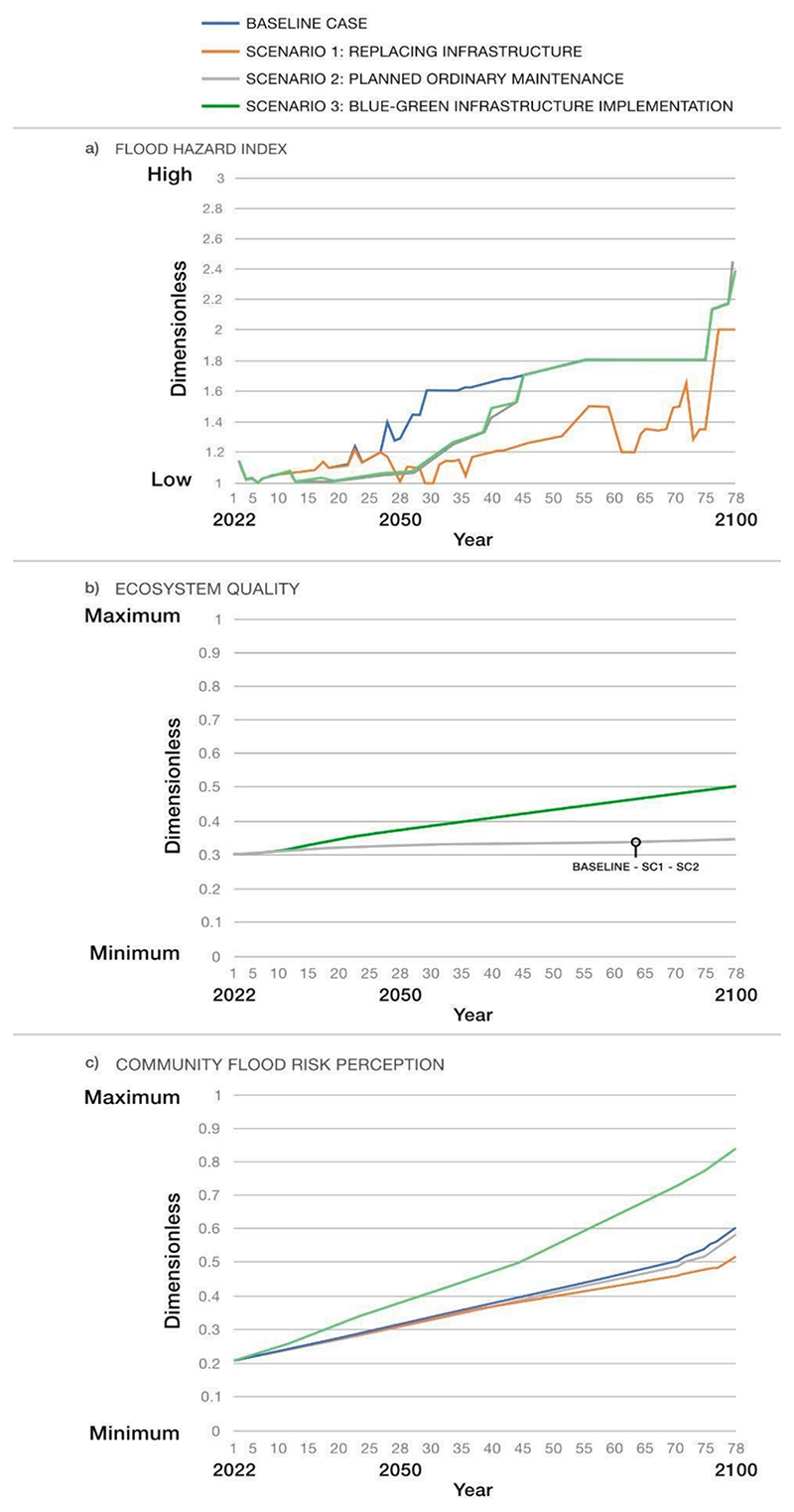
a) ‘flood hazard index’, b) ‘ecosystem quality’ and c) ‘community flood risk perception’ outputs under scenarios 1–3. where scenarios overlap, their labels are separated by a hyphen (–).

**Fig. 7:**
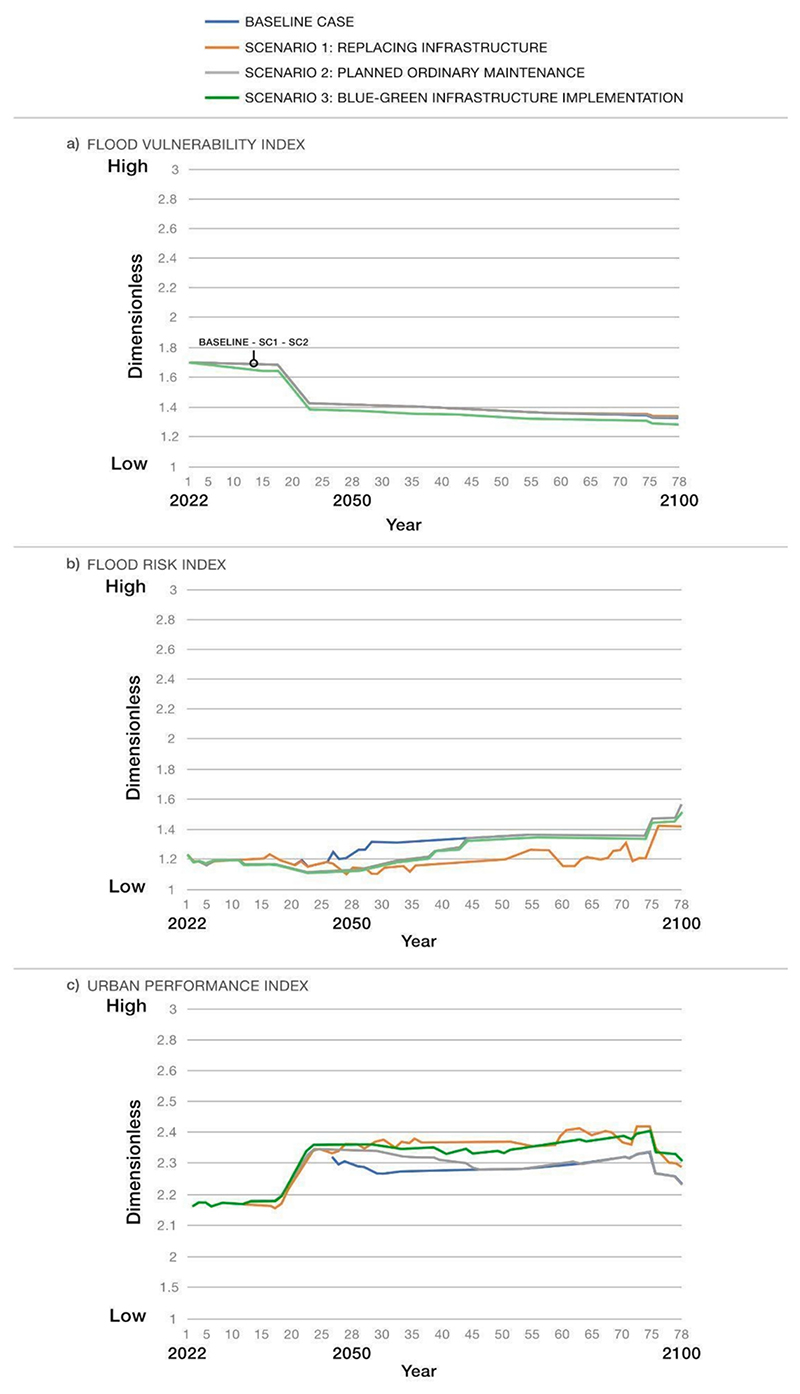
a) ‘flood vulnerability index’, b) ‘flood risk index’ and c) ‘urban performance index’ outputs under scenarios 1–3. where scenarios overlap, their labels are separated by a hyphen (–).

For the sake of brevity, only the most relevant model outputs resulting from scenario comparison are described in this work. These outputs are presented below by some of the representative variables of the system, namely ‘flood hazard index,’ ‘ecosystem quality state,’ ‘community flood risk perception,’ ‘flood vulnerability index,’ ‘flood risk index,’ and ‘urban performance index’) (see Figs. 6 and 7). The outputs are in dimensionless units and range from 1 (low) to 3 (high) if they are indices, and from 0 (minimum level) to 1 (maximum level) if they are co-benefits. It is worth mentioning that the focus of the SF model is on trends (and underlying reasons influencing them) and on the comparative analysis of variable states under different conditions. Therefore, although the results show minor differences in some variables across scenarios, the underlying mechanisms driving these trends vary among scenarios.

#### 4.1.1 ‘Flood hazard index’ (see Fig. 6a)

The baseline case indicates a growing flood hazard over time, mainly due to issues in grey systems (i.e., stormwater and drainage systems) caused by sediment build-up and system ageing. The rapid increase of the flood hazard around 2090 can be attributed to the deterioration of the Thames Defences. Overall, Scenario 1, which involves replacing grey infrastructure at the end of its service life, seems to be the most effective in reducing flood hazards compared to the baseline. Regular maintenance of grey systems in Scenario 2 and the implementation of BGI in Scenario 3 both decrease the ‘flood hazard index’ over about ten years compared to the baseline. Maintenance of grey systems means fewer sediments accumulate, increasing their storage capacity and reducing their weir. Meanwhile, BGI implementation eases stress on grey systems, leading to less water flooding from urban systems, extending the life of grey systems, and ultimately reducing the ‘flood hazard index’ *(interaction between BGI, hydrological sub-system and grey infrastructure)*. However, when the service life of the grey systems is over (approximately in 2070), the BGI alone (Scenario 3) is insufficient to reduce the ‘flood hazard index’ compared to the baseline. Therefore, it is crucial to analyse other scenarios involving additional long-term measures, both BG and grey.

#### 4.1.2 ‘Ecosystem quality’ (see Fig. 6b)

In Scenarios 1 and 2, the ‘ecosystem quality’ shows no major differences from the baseline. However, in Scenario 3, the implementation of BGI increases the ‘ecosystem quality’, likely due to the growth of ‘green spaces experience’ *(interaction between BGI and social sub-system)* and biodiversity *(interaction between BGI and environmental sub-system)*.

#### 4.1.3 ‘Community flood risk perception’ (see Fig. 6c)

In Scenario 1, ‘community flood risk perception’ decreases compared to the baseline. This may be because the replacement of grey systems affects the social and economic sub-systems. In particular, it contributes to an increased sense of safety within the community *(interaction between grey infrastructure and social sub-system)* and a general reduction in tangible damage to the built environment from flooding *(interaction between grey infrastructure and economic sub-system)*. On the contrary, in Scenario 3, community perception improves compared to the baseline mainly as the implementation of BGI provides a greater involvement of citizens and thus a greater awareness of the flood risk in the area *(interaction between BGI and social sub-system)*.

#### 4.1.4 ‘Flood vulnerability index’ and ‘flood risk index’ (see Fig. 7a and 7b)

The ‘flood vulnerability index’ (see Fig. 7a) does not show significant changes in Scenarios 1 and 2 compared to the baseline. The decreasing trend of flood vulnerability mainly depends on the positive effect of the regeneration plan on the quality of the built environment, including buildings, transport services, and infrastructure. In Scenario 3, the increase of co-benefits (such as ‘residents’ well-being,’ ‘ecosystem quality,’ ‘community flood risk perception’) due to BGI implementation *(interaction between BGI and social/environmental sub-systems)* contributes to an additional decrease in vulnerability. The limited change of the ‘flood vulnerability index’ over time across scenarios indicates that the ‘flood risk index’ (see Fig. 7b) is more influenced by changes in the ‘flood hazard index’.

In the long run, the ‘flood risk index’ generally tends to increase. However, BGI implementation, as depicted in Scenario 3, demonstrated a notable decrease in flood risk over approximately ten years compared to the baseline. This reduction can be attributed not only to the hydrological benefits of BGI when coupled with functioning grey infrastructure (see ‘Flood hazard index’ section) but also to the enhanced co-benefits (such as ‘residents’ well-being,’ ‘ecosystem quality,’ ‘community flood risk perception’) *(interaction between BGI and social/environmental/hydrological sub-systems)*. As long as the grey systems function properly, the flood risk is reduced in all the scenarios (especially in Scenario 1) compared to the baseline.

#### 4.1.5 ‘Urban performance index’ (see Fig. 7c)

The trend of the ‘urban performance index’ does not change across scenarios until around 2030, which corresponds to the implementation of the management measures. From 2030 onwards, urban performance improves in all scenarios compared to the baseline. However, while conditions in Scenario 2 become comparable to the baseline around 2065, Scenarios 1 and 3 are more desirable in the long term. This is mainly because of the enhanced effectiveness of flood mitigation infrastructure in Scenario 1 *(interaction between grey infrastructure and hydrological sub-system)* and the increase in both flood mitigation infrastructure and co-benefits in Scenario 3 *(interaction between BGI and social/environmental/hydrological sub-systems)*.

### 4.2 Scenario combination

**Fig. 8:**
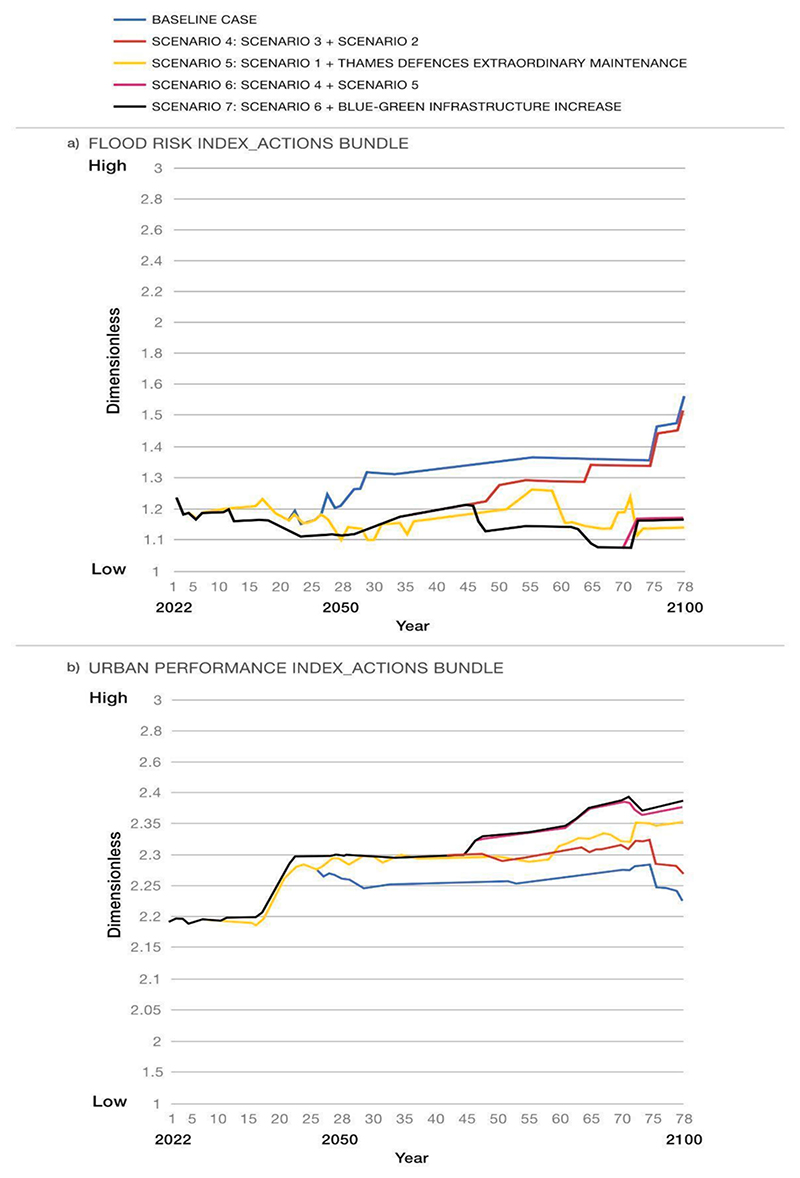
a) ‘flood risk index’, b) ‘urban performance index’ outputs under scenarios 4–7.

By combining the actions proposed in Scenarios 1–3 over time, some bundles of actions were developed and analysed. For the sake of brevity, only the model outputs for the variables ‘flood risk index’ and ‘urban performance index’ (see Fig. 8) are presented below.

The comparison of scenarios revealed that the most effective options are Scenarios 6 and 7, where the implementation of BGI along with ordinary and extraordinary maintenance of grey systems is planned. Despite the doubling of BGI areas, there is no significant change in the behaviour of the ‘flood risk index’ and ‘urban performance index’ in Scenario 7 compared to Scenario 6. This confirms the crucial role of well-functioning grey systems in mitigating flood risks, indicating that implementing BGI alone, despite its multiple benefits, would not be sufficient to control surface runoff. Moreover, the minimal variation between Scenarios 6 and 7 suggests that the designated BG areas in the regeneration plan (used in Scenario 6) are sufficient for reducing flood risk and enhancing the resilience of the urban system to flooding. Consequently, there seems to be no necessity to invest in expanding the BG areas.

### 4.3 Sensitivity analysis

**Fig. 9:**
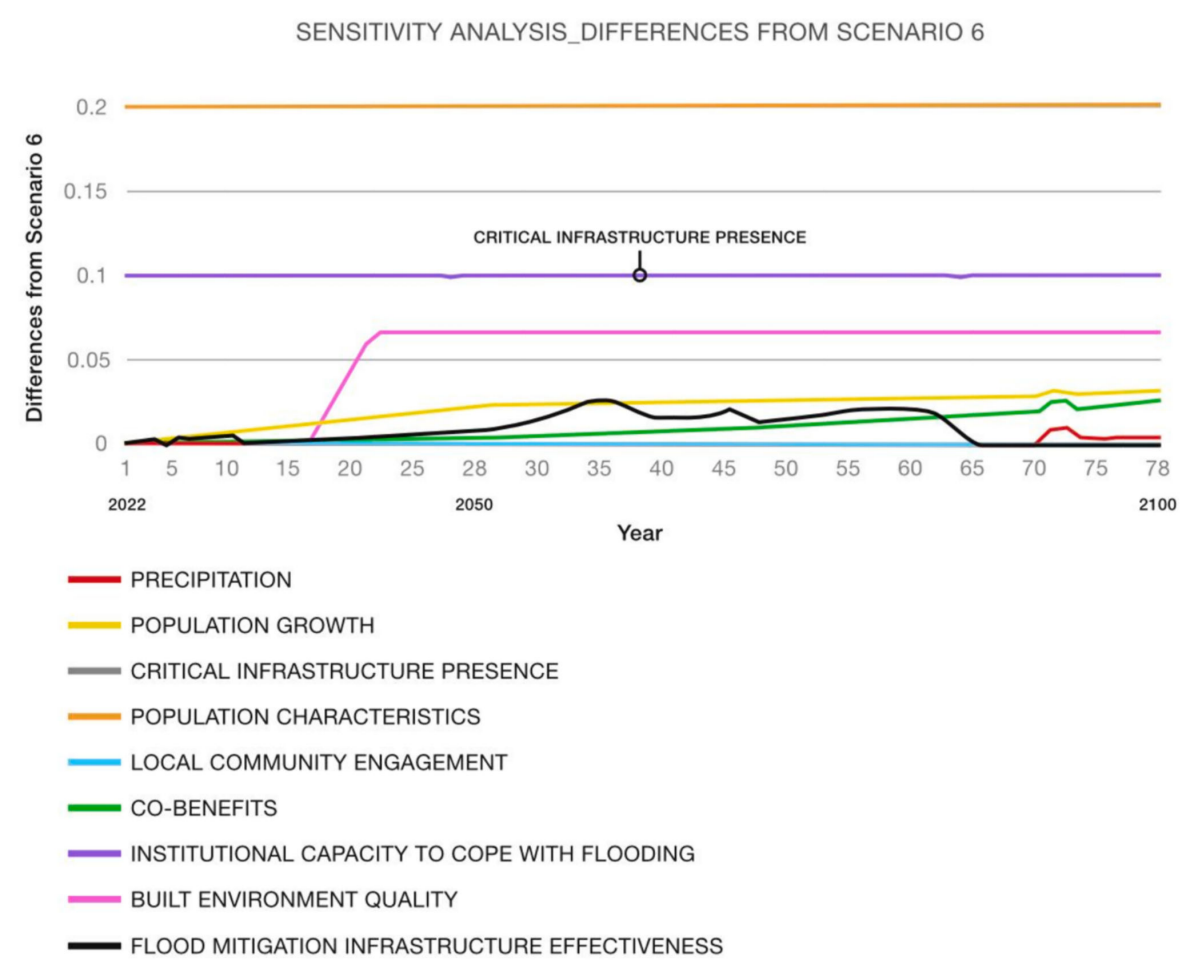
Sensitivity analysis related to ‘urban performance index’. The differences between Scenario 6 and single parameters variation are represented.

A sensitivity analysis was performed to investigate the influence of single variables of the model on the ‘urban performance index’. For the sake of brevity, Fig. 9 shows the impact of each parameter on flood resilience, focusing on Scenario 6, which is deemed the most effective scenario. The variables that were altered during the sensitivity analysis include: ‘precipitation,’ ‘population growth,’ ‘critical infrastructure presence (i.e., hospitals, schools, electricity sub-stations),’ ‘population characteristics (i.e., elderly and children),’ ‘local community engagement,’ ‘co-benefits (i.e., ecosystem quality, residents’ well-being, attractiveness of local area and community flood risk perception),’ ‘institutional capacity to cope with flooding,’ ‘built environment quality,’ and ‘flood mitigation infrastructure (i.e., stormwater and drainage systems and tidal river defences)’.

‘Population characteristics,’ ‘critical infrastructure presence,’ and ‘institutional capacity to cope with flooding’ appear to be the most influential variables affecting the ‘urban performance index’. This suggests that the strategy outlined in Scenario 6, which already addresses co-benefits and the effectiveness of flood mitigation infrastructure, could be strengthened by implementing measures targeting these specific aspects such as ‘population characteristics,’ ‘critical infrastructure presence’ and ‘institutional capacity to cope with flooding’. This enhancement aims to further improve Thamesmead resilience to flooding in Scenario 6.

## 5 Discussion

This section discusses to what extent the proposed modelling approach contributes to the socio-hydrology field by providing an answer to the research questions raised in Section 1.

### 5.1 The role of System Dynamics modelling in offering a holistic understanding of complex socio-hydrological systems and interactions

This work has shown how quantitative SD modelling can be used for building a holistic picture of the complex interconnections that involve socio-hydrological systems, such as urban systems prone to flood risk, particularly focusing on the role of BGI. Compared to traditional hydrological modelling approaches and tools for flood risk management (see e.g., Montanari et al., 2013; Di Baldassarre et al., 2015; Sivapalan and Blöschl, 2015), SD simulation modelling offers detailed insights into the implications of flood risk and BGI implementation (including the multiple co-benefits they can generate for the community) in an urban area, thereby improving understanding and decision-making.

The adoption of a system-based approach allowed for the integration of different sub-systems addressing hydrological, social, economic, and environmental factors influencing and influenced by flood risk. More specifically, the main dynamics of the system were isolated into thematic sub-models (e.g., ‘flood hazard’ sub-model, ‘co-benefits analysis’ sub-model, ‘tangible damage evaluation’ sub-model, etc.) and analysed individually, while providing a clear picture of the entire system and revealing complex interactions across scales and sectors.

On one hand, the construction of an SD simulation model made it possible to consider the bidirectional feedbacks between flood and urban dynamics overcoming the assumption of stationarity of the social component, typical of traditional hydrological models. For example, the model incorporated the impact of population growth on building development and consequently on soil imperviousness (see e.g., ‘Flood hazard’ sub-model described in Section 3.3). At the same time, the influence of soil imperviousness on flood hazards was explored, along with how these hazards, in turn, affect some population-related aspects, such as the well-being of residents and the attractiveness of the area (see e.g., the effect that flood depth has on ‘Tangible damage evaluation’ and ‘Co-benefits analysis’ sub-models in Section 3.3). On the other hand, the ability of the SD simulation model to analyse not only quantitative (‘hard’ or physical) but also qualitative (‘soft’ or intangible) aspects enabled the development of sub-models that would be challenging to interpret without considering these qualitative elements (see e.g., ‘Flood vulnerability’ sub-model described in Section 3.3). Furthermore, the evaluation of the potential of BGI in terms of risk reduction, together with co-benefits and increase in urban flood resilience, demonstrated how the quantitative SD model overcomes the limited inclusion of these elements in existing frameworks (see e.g., Kabisch et al., 2016; Calliari et al., 2019).

The scenario analysis then helped assess the impact of different resilience-enhancing actions on representative variables of the system. To this aim, the development of indices summarising the complex information available in the model (such as ‘flood hazard index,’ ‘flood vulnerability index,’ ‘urban performance index’) supported in providing simple yet robust insights into modelling results. Effective strategies were identified and their effectiveness, potential consequences, side effects and synergistic effects were modelled and visualised. Therefore, scenario analysis allowed the evaluation of the feasibility and relevance of the selected strategies in view of the main objective (i.e., managing flood risk and improving urban flood resilience), while understanding the multi-dimensional implications they might have (i.e., their interaction with different sub-systems) (see Section 4.2 for further details). For example, through the analysis of hazard, vulnerability, risk, and urban performance indices, it was observed that coupling BGI with planned extraordinary and ordinary maintenance of grey systems emerges as a promising long-term strategy for maximizing benefits and co-benefits to the system. The interactions between different sub-systems, such as environmental, social, economic, and hydrological factors, played a crucial role in shaping flood risk within the urban area. For instance, the replacement of grey infrastructure affects both the social and economic sub-systems, contributing to an increased sense of safety within the community and reducing tangible damage to the built environment from flooding. Similarly, the implementation of BGI provides co-benefits such as improved ecosystem quality and increased community flood risk perception, which contribute to a decrease in flood vulnerability and overall flood risk. These interactions highlight the interconnected nature of flood risk dynamics and underscore the importance of considering multiple factors in flood risk management strategies.

Lastly, sensitivity analysis helped identify the most influential factors for achieving flood resilience. To provide an example, in the case of Scenario 6, which is the most effective and already includes different types of flood infrastructure, aspects such as ‘population characteristics,’ ‘critical infrastructure presence’ and ‘institutional capacity to cope with flooding’ may have a relevant impact on system resilience. Therefore, additional strategies targeting these specific aspects should be considered to further increase system resilience to flooding in Scenario 6.

In conclusion, in this application, SD modelling offers a comprehensive understanding of complex socio-hydrological systems and their dynamics, contributing to informed decision-making in long-term flood risk management and enhancing urban flood resilience. In particular, the analysis of interactions between different sub-systems, coupled with the evaluation of BGI’s potential for risk reduction and the generation of co-benefits, offers decision-makers actionable insights into the multifaceted nature of flood risk and resilience. Moreover, the visualization of modelling results through indices facilitates the communication of complex information, supporting stakeholder engagement and consensus-building processes.

### 5.2 The role of scientific and stakeholder knowledge combination within System Dynamics modelling for increasing urban flood resilience

Compared to the existing literature which often overlooks the importance of stakeholder engagement in socio-hydrological modelling due to e.g., time and funding constraints, and the lack of trust that decision/policy-makers have in participatory approaches, this work demonstrates how an iterative combination of scientific data and stakeholder knowledge provided several benefits that would not be achievable through scientific knowledge alone. Engaging stakeholders through participatory modelling techniques, such as workshops and interviews, served a dual purpose. It provided both local knowledge for tailoring the model to the study area and expert insights for supporting understanding and quantification of the socio-hydrological dynamics that are limitedly explored in the scientific literature. More specifically, stakeholder contribution was necessary for i) identifying key variables and mechanisms of the complex urban system, ii) collecting data to build the quantitative SD model, and iii) validating specific inputs and outputs of the model. The multi-step process of knowledge gathering and structuring in the form of an SD model made it possible to quantify elements of the urban system that are beyond the scope of traditional hydrological models (such as ‘residents’ well-being’, ‘community flood risk perception,’ ‘ecosystem quality,’ etc.) providing a better insight of how different factors interact within an urban system influencing flood risk and resilience. The BOT graphs drawn by stakeholders during the qualitative modelling phase (refer to Coletta et al., 2024 for further details) were crucial in defining the initial value of the co-benefits in the model and then simulating their trend (see Section 3.3). However, as highlighted in Section 3.1, the flexible nature of the proposed modelling approach would allow for the development of BOT graphs with stakeholders directly during the quantitative modelling phase, if necessary. In addition, stakeholders enabled the development of effective scenarios for managing flood risk and enhancing resilience to floods. Specifically, they supported the modeller in selecting actions (mainly BGI) and strategies to be implemented in the SD model based on the wide range of objectives and investments foreseen in the regeneration plan. This helped build a model that better reflects local conditions, interests and perceptions, ultimately providing relevant information to decision-makers.

### 5.3 The role of Blue-Green Infrastructure – and their interplay with grey infrastructure – in increasing urban flood resilience by interacting with different sub-systems

This work has also contributed to the assessment of the multi-dimensional impacts of BGI on urban flood resilience. The scenario analysis confirmed, in line with recent literature, the ability of BGI to provide not only hydrological benefits (mainly about the reduction of surface runoff) but also multiple social and environmental benefits (i.e., the co-benefits) which are often even more relevant. For example, the implementation of different BGI (such as wetlands, blue/green roofs, woodlands) may increase the ‘ecosystem quality’ and the ‘residents’ well-being’ thanks to the possibility of more ‘green spaces experience’. Besides that, their development asks for more ‘local community engagement’ thus improving the ‘community perception of flood risk’ (see Section 3.3 and Section 4.1). Nevertheless, the model also showed that the implementation of BGI would not be sufficient on its own to both reduce flood risk and enhance urban flood resilience. For this reason, the combined effects of the implementation of BGI and existing well-functioning grey systems were examined (refer to Section 4.2). From a hydrological point of view, BGI extends the service life of ageing stormwater and drainage systems reducing the quantity of ‘surface runoff’ and sediments they have to manage. Therefore, the BGI resilience-enhancing ability was confirmed by the support provided by the Hybrid infrastructure (integrated grey and Blue and Green solutions) in supporting urban systems, namely adapting to the increasing threat of flooding, while also providing environmental, social, and economic co-benefits. In further evidence of this assertion, the scenario analysis demonstrated an increase in the ‘urban performance index’ (i.e., urban resilience measure in this work) when BGI is implemented along with existing grey measures (see Section 4.2).

## 6 Main challenges and limitations

Modelling challenges were mainly related to the connection between technical variables (e.g., ‘tangible damage due to flooding’) and ‘soft’ variables (e.g., ‘community flood risk perception’), as well as some data collection (e.g., of co-benefits). Some simplifications in the quantification of variables were therefore introduced. For example, co-benefits were expressed in dimensionless terms, measured on a scale from 0 to 1 (or as a percentage), where 0 corresponds to the minimum level of the co-benefit, and 1 to the maximum level. However, further consultations with stakeholders and targeted interviews helped deal with the lack of some data. Despite the fundamental contribution of stakeholders, the time and workload needed for the organisation of interviews and workshops (as in every participatory activity) increased the time needed for model building. However, co-developing models with stakeholders had a huge benefit related to the amount of expert knowledge that could be included in the model. Furthermore, arranging online meetings that could meet the needs of all stakeholders limited the level of interaction among them. Nevertheless, online meetings allowed activities to continue during the COVID-19 pandemic.

Effectively communicating the purpose and key characteristics of the model to stakeholders was challenging, especially as SD models are not always straightforward (as mentioned by Meadows, 2008). For this purpose, sharing some briefing notes on the model before meetings helped support a better comprehension and a more effective contribution to activities. In general, stakeholder feedback on the content of the workshops was positive; particularly, they appreciated the opportunity to reflect on shared interests in the area and to be informed on the perspectives of other stakeholders. The coding of the interviews and workshops also proved time-consuming. Stakeholders shared a lot of information with the modeller, requiring an iterative approach to identify, select, and validate relevant information.

As SD models have been initially developed to investigate the temporal dimension in non-spatial systems, the lack of an explicit representation of spatial processes is a key constraint of SD modelling (see e. g., Voinov et al., 2018). There are, however, different ways of including spatial information in SD models which include the conceptualization of phenomena in an aggregated form (such as, for the present application, a simple water balance). As the purpose of the work is, ultimately, to propose a global assessment for the area based on the combination of different aspects (hydrological, social, environmental) and indices, we believe that the lack of an explicit spatial dimension is not a major limitation for the present work. However, future modelling efforts will be definitely oriented to better detail some dynamics at the spatial level through e.g., the use of semi-distributed information or the combination of the SD model and spatially distributed modelling approach. In addition, although the SD model can inform decision-makers at a planning/strategic level, it has limited applicability in the analysis of individual or micro-scale dynamics, which could be useful at other stages of the design process. Furthermore, a thorough analysis and comparison of strategies in terms of benefits and costs have to be supported by other methodologies, such as Cost-Benefit Analysis and Multiple-Criteria Decision Analysis (see e.g., Belton and Stewart, 2002). The need to combine SD modelling with other decision support approaches should therefore be considered.

Even if the current structure of the model is adequate for the analysis presented, future developments should further explore processes such as the relationship between the effects of climate change and the reduction of green areas, the decrease in the value of private property due to flooding damage, and the distinction between the attractiveness of the area for residents and investors.

## 7 Conclusions

The effectiveness of traditional hydrological modelling for flood risk management in urban areas is limited because they often focus on purely hydrological issues, neglecting the interaction between hydrology and key elements of the urban system (e.g., social, environmental, economic) and, therefore, affecting the effectiveness of flood risk reduction strategies. In this context, socio-hydrological modelling aims at integrating the information provided by traditional hydrological models in a broader system picture that – in this work – was effectively built using System Dynamics (SD) modelling. The adoption of a socio-hydrological modelling approach based on SD proved useful for a twofold reason: i) to better understand the multiple implications of urban and social dynamics on flood risk (which is therefore not seen as static); ii) to describe the multi-dimensional impacts associated to the implementation of flood risk mitigation measures (such as Blue-Green Infrastructure – BGI), which include both flood risk reduction potential and the generation of multiple co-benefits for the urban communities and environment. Ultimately, this work outlined how BGI can contribute to enhancing the resilience of urban systems in the face of flood risk while considering the numerous implications within a highly dynamic urban context. The proposed modelling approach benefited significantly from the active involvement of local stakeholders, considering the knowledge they can provide on the area, as well as their perception of the impacts of flood risk and BGI implementation. Specific reference was made to one of the case studies of the CUSSH and CAMELLIA projects, namely Thamesmead (London, UK), perceived as being increasingly vulnerable to flooding. However, the developed modelling approach is suitable for replication in other contexts.

## Supplementary Material

**Appendix A. Supplementary data**

Supplementary data to this article can be found online at https://doi.org/10.1016/j.jhydrol.2024.131248.

Supplementary Material

## Figures and Tables

**Table 1:** The proposed participatory modelling approach. The activities with their objectives, the tools/methods adopted, and the expected results are shown. The five steps of the qualitative modelling phase are in grey, while the three steps of the quantitative modelling phase are in white. This work focuses on the quantitative phase. For more details on the qualitative one, please refer to Coletta et al. (2024).

#	TASKS	AIMS	TOOLS/METHODS	OUTCOMES
1	Literature review and baseline analysis for preliminary Causal Loop Diagram (CLD) building	To build a preliminary CLD, based on the scientific knowledge and background information on the study area	– Literature review on urban flooding – Gathering information about the study area, e.g., from reports, existing models, etc.	A preliminary CLD on the study area, based on the scientific knowledge, focused on urban flood risk
2	Interviews with stakeholders for preliminary CLD improvement	To collect and structure stakeholder knowledge for improving the key cause-effect chains of the preliminary CLD	– Semi-structured interviews with stakeholders and email exchange – Analysis of semi-structured interviews – Integration of scientific and stakeholder knowledge	A CLD on urban flood risk which integrates scientific and stakeholder knowledge
3	CLD causal structure validation	To validate the general structure and key CLD connections	Collective model testing and participatory exercises	Final structure of CLD
4	Behaviour Over Time (BOT) graphs construction with stakeholders	To collect stakeholder perception on the dynamic evolution of some key variables of the system	BOT graphs construction	Graphs on the dynamic evolution of the system based on stakeholder perception
5	CLD integration based on stakeholder-built BOT graphs	– To analyse the main dynamics and impacts of flood in the CLD – To integrate BOT graphs results into the final CLD	BOT graphs and key CLD feedback loops analysis	Formulation of hypotheses on urban system dynamics and flood risk management policies
1	Building a Stock and Flow (SF) model related to the current system condition	– Identifying stock, flow, auxiliary and input variables – Determining equations and parameters using literature, existing models, reports, databases, and stakeholder input – Developing sub-models and calculating indices that provide aggregated information on system dynamics	– Literature review and data collection for model input, e. g., from reports, existing models, etc. – Interviews with stakeholders and email exchange – Behaviour Over Time (BOT) graphs	A SF model on urban flood risk combining scientific and stakeholder knowledge
2	Validating the SF model	Validating the model through analysis of key variables/indices over time	– Comparing stakeholder-built BOT graphs with model-simulated trends for the same variables – Collective model testing and participatory exercises to validate other variables	Final SF model on urban flood risk
3	Building and analysing future scenarios	– Developing future scenarios for stakeholder discussion – Co-designing alternative scenarios with stakeholders – Comparing future scenarios – Selecting the most effective scenario and performing a sensitivity analysis to identify key monitoring variables	– Reviewing literature and engaging stakeholders in scenario exploration – Comparative analysis of urban system dynamics under different flood scenarios – Sensitivity analysis of the most effective scenario	Recommendations for implementing an effective strategy (bundle of actions) and monitoring key variables to enhance urban flood resilience

**Table 2:** List of the stakeholders involved in the modelling process.

STAKEHOLDERS	ORGANIZATIONS	ROLES
Stakeholder 1	Housing Association/Developers	Director
Stakeholder 2	Housing Association/Developers	Head of Landscape & Placemaking
Stakeholder 3	Local Authority	Flood Risk and Development Manager
Stakeholder 4	Local Authority	Project Manager
Stakeholder 5	Local Authority	Manager of Operations
Stakeholder 6	Environmental Non-Governmental Organization	Senior Manager
Stakeholder 7	Company of consulting and engineering/architectural design	Director
Stakeholder 8	Company of consulting and engineering/architectural design	Catchment Partnership Development Officer
Stakeholder 9	Local nature conservation charity	Conservation ecologist

**Table 3:** Scenario description.

SCENARIO	DESCRIPTION
Baseline Scenario	This scenario described the most likely evolution of the system if the main input variables (e.g., ‘precipitation’, ‘evapotranspiration,’ ‘sea level rise,’ ‘infrastructure quality,’ ‘institutional capacity to cope with flooding’) change according to the climate change projections and the regeneration plan for Thamesmead (TM).
Scenario 1 – ‘Replacing infrastructure’	In this scenario, stormwater and drainage systems were replaced at the end of their service life (approximately 40 years). Therefore, changes in the parameters of these systems were made in 2046 and 2087.
Scenario 2 – ‘Planned ordinary maintenance’	In this scenario, stormwater and drainage systems were regularly maintained from 2030 onwards. The effects of periodically cleaning the systems and the subsequent extension of their service life (about 10 years) were evaluated.
Scenario 3 – ‘Blue-Green Infrastructure (BGI) implementation’	In line with the regeneration plan for TM, this scenario examined the role that BGI can play in addressing flooding and improving co-benefits (e.g., ‘ecosystem quality’ and ‘residents’ well-being’). The hydrological benefit of BGI measured through surface runoff reduction and biodiversity performance was implemented from 2030 onwards. Specifically, intensive BG roofs, urban green avenues/woodlands, wetlands, parks, and lakes and canals re-naturalisation were introduced.

**Table 4:** Description of combined scenario.

SCENARIO	DESCRIPTION
Scenario 4 – ‘BGI implementation + planned ordinary maintenance’	This scenario proposes the implementation of BGI from 2030 (as in Scenario 3) and ordinary maintenance actions from 2050 (as in Scenario 2).
Scenario 5 – ‘Replacing infrastructure + Thames Defences extraordinary maintenance’	This scenario proposes the replacement of the stormwater and drainage systems at the lifecycle end in 2046 and 2087 (as in Scenario 1) and the modification of the Thames Defences around 2090.
Scenario 6 – ‘Scenario 4 + Scenario 5’	This scenario suggests the implementation of the bundle of actions of Scenario 4 and, from 2070 onwards, that of Scenario 5.
Scenario 7 – ‘Scenario 6 + BGI increase’	This scenario proposes the implementation of the same actions as Scenario 6 while doubling the areas of the BGI.

## Data Availability

Data will be made available on request.
