# Supplementary material for: Socio-hydrological modelling using participatory System Dynamics modelling for enhancing urban flood resilience through Blue-Green Infrastructure
Source: J Hydrol (Amst). Author manuscript; Available in PMC 2024 Oct 16. (PMC7616713; doi:10.1016/j.jhydrol.2024.131248)
Supplement: Supplementary Material [file EMS199300-supplement-Supplementary_Material.docx]

Socio-hydrological modelling using participatory System Dynamics modelling for enhancing urban flood resilience through Blue-Green Infrastructure

**Supplementary Material**

This section includes additional details related to (1) the workshop agenda on the model validation and scenarios design, (2) mathematical equations, initial values and data sources behind the model, (3) values of the changed variables in the different scenarios.

**Supplementary Material Section 1.** Workshop agenda of Thamesmead SF model validation and scenarios design with stakeholders.

Date: 27/10/2022

Time: 2h30

Location: online, using Microsoft Teams

Aims*:*

- To briefly present the flood risk SD simulation model
- To validate specific inputs/outputs of the model
- To show and discuss the developed future scenarios impacting key system elements
- To discuss other factors/measures that may contribute to future scenarios

| **TIME** | **ACTIVITY** | **OBJECTIVE** |
| --- | --- | --- |
| 10 min | Welcome and introduction | Warm up for orientation and goals clarification |
| 15 min | Flood simulation model presentation | -Summary of the modelling process  -Presentation of the simulation model  -Preparation of participants for the next activities |
| 45 min | Validation of specific inputs/outputs of the model in the current condition (semi-structured interview style) | To reach a consensus on the model |
| 40 min | Discussion on the developed future scenarios (semi-structured interview style) | Reaching a consensus on the developed future scenarios and their influencing factors/measures |
| 20 min | Design of possible other scenarios to be tested afterwards  (semi-structured interview style) | -Elicitation of stakeholders’ ideas for scenarios to be tested that could include specific problem-framing  -Knowledge expansion |
| 10 min | Evaluation  Next steps and closing |  |

**Supplementary Material Section 2.** Mathematical equations, initial values, and data sources behind the SF model.

Initial Time: 1 (01/01/2022)

Final Time: 28835 (31/12/2100)

Time Step: 1

Units for time: Day

| **VARIABLE** | **VARIABLE TYPE** | **EQUATION OR VALUE** | **INITIAL**  **/CONSTANT VALUE** | **DATA AND COMMENTS** | **DATA SOURCES** |
| --- | --- | --- | --- | --- | --- |
|  |  | **LAND CONSUMPTION SECTION** |  |  |  |
| RESIDENTIAL DENSITY GROWTH RATE DUE TO POPULATION GROWTH  *(dwellings/(Day*ha))* | Lookup | [(1,0)-(22266,0.003)], (1,0),(1460,0.0028), (3285,0.0028), (5110,0.0028), (6935,0.0028), (8760,0.0028), (10950,0), (12775,0), (14600,0), (16425,0), (18250,0), (20075,0), (21900,0), (23725,0), (25550,0), (27375,0) |  | Between 2020 and 2050, the population of Thamesmead is projected to increase from 40000 to 100000.  100000/40000 = 2.5  Given this population growth, particularly the increase in the number of households, we anticipate a corresponding rise in housing construction. As a result, the ratio between the current residential density and the projected 2050 residential density will also be 2.5. By multiplying the current residential density (20.7 - as cited in Hall and Madden (2018), Page 11)) by 2.5, we derive the future residential density, which amounts to 51.7.  51.7 (final residential density) - 20.7 (initial) = 31.05 dwellings/hectare  Subsequently, dividing this difference (31.05) by the 10,950 days (30 years) over which the population is expected to increase yields the daily growth rate of residential density, which is 0.0028.  We assumed that this daily growth rate would remain constant over the next two decades (2050-2070), mirroring the rate observed from 2020 to 2050 | -Mulder, 2006  -Landcom, 2011  -Hall and Madden, 2018  -Peabody, 2021 |
| fraction of residential density growth  *(dwellings/(Day*ha))* | Auxiliary | RESIDENTIAL DENSITY GROWTH RATE DUE TO POPULATION GROWTH (Time) |  |  |  |
| density of building development increase rate  *(dwellings/(Day*ha))* | Inflow | fraction of population growth |  |  | -Landcom, 2011 |
| Density of Building Development  *(dwellings/ha)* | Stock | density of building development increase rate | 20.7 |  | -Hall and Madden, 2018 |
| DENSITY APPROXIMATION FOR IMPERVIOUSNESS  *(ha/dwellings)* | Constant |  | 1 | The imperviousness percentage, being dimensionless, can be approximately correlated with the density of building development. In order to remove the units associated with building density, we opted to introduce a variable with units that nullify the building density | -Butler et al. 2018 |
| STATISTICAL COEFFICIENT  (Dmnl) | Constant |  | 6.4 |  | -Butler et al. 2018 |
| percentage imperviousness  (Dmnl) | Auxiliary | STATISTICAL COEFFICIENT*SQRT(Density of Building Development*DENSITY APPROXIMATION FOR IMPERVIOUSNESS) |  | According to Butler et al. (2018), imperviousness percentages typically fall within the range of 25 to 100. In London, an imperviousness percentage of approximately 35% is associated with open housing, whereas a percentage closer to 55% is typical for dense housing. Given the projected doubling of housing in Thamesmead by 2050, it is plausible to anticipate that by 2070, the imperviousness percentage will approach 55% | -Butler et al. 2018 |
| PERCENTAGE BASE VALUE  (Dmnl) | Constant |  | 100 |  |  |
| imperviousness coefficient  (Dmnl) | Auxiliary | percentage imperviousness/PERCENTAGE BASE VALUE |  |  |  |
|  |  | **WATER BALANCE SECTION** |  |  |  |
| PRECIPITATION  *(mm/Day)* | Data |  | Precipitation data as a timeseries  Specificare excel | Average daily rainfall data from 2010 to 2070 for Thamesmead were derived due to the absence of monitoring stations within Thamesmead itself. The historical daily precipitation records from the four nearest monitoring stations to Thamesmead were utilized (https://nrfa.ceh.ac.uk/data/search). Inverse Distance Weighting (IDW) was employed to interpolate the precipitation data for Thamesmead. The weights assigned in the IDW process are directly proportional to the distances of the stations from the Thamesmead area. The UK Climate Projections (UKCP09) downscaled from the Met Office regional climate model were implemented in both precipitation and evapotranspiration timeseries. The highest changes in both the annual mean precipitation and potential evapotranspiration, considering a 90% probability level under a high-emission future scenario, projected for the 2080s relative to the baseline period of 1961–1990, were considered and distributed to the daily time scale (see Murphy et al. 2009 and Thompson, 2012). To include the probabilistic component in the hazard assessment, the precipitation dataset was manually modified implementing several events with 2, 5, 10, 30, 50, 100 and 500-year future return periods. For this purpose, the 24-hour cumulated rainfall Intensity Duration Frequency curves for the City of London under changing climatic conditions were used (see Prodanovic and Simonovic, 2007). | -Murphy et al. 2009  -Coxon et al. 2020  -https://nrfa.ceh.ac.uk/data/search  -Prodanovic and Simonovic, 2007 |
| FULL IMPERVIOUSNESS COEFFICIENT  *(Dmnl)* | Constant |  | 1 |  | -Lemaire et al. 2021 |
| precipitation’s pervious component  *(mm/Day)* | Auxiliary | PRECIPITATION*(FULL IMPERVIOUSNESS COEFFICIENT-imperviousness coefficient) |  |  | -Lemaire et al. 2021 |
| precipitations’ impervious component  *(mm/Day)* | Auxiliary | PRECIPITATION*imperviousness coefficient |  |  | -Lemaire et al. 2021 |
| DESIGN SOIL WETNESS INDEX  *(Dmnl)* | Constant |  | 0.45 |  | -Butler et al. 2018 |
| infiltrating flow  *(mm/Day)* | Auxiliary | precipitation’s pervious component*(FULL IMPERVIOUSNESS COEFFICIENT-SOIL WETNESS INDEX) |  |  | -Lemaire et al. 2021 |
| overland flow  *(mm/Day)* | Auxiliary | precipitation’s pervious component* SOIL WETNESS INDEX |  |  | -Lemaire et al. 2021 |
|  |  | **GROUNDWATER LEVEL SECTION** |  |  |  |
| EVAPOTRANSPIRATION  *(mm/Day)* | Data |  | Evapotranspiration data as a timeseries | Average daily potential evapotranspiration data from 2010 to 2070 for Thamesmead were derived using a methodology due to the absence of monitoring stations within Thamesmead itself. The historical daily potential evapotranspiration records from the four nearest monitoring stations to Thamesmead were utilized (https://nrfa.ceh.ac.uk/data/search). Inverse Distance Weighting (IDW) was employed to interpolate the potential evapotranspiration data for Thamesmead. The weights assigned in the IDW process are directly proportional to the distances of the stations from the Thamesmead area.  The UK Climate Projections (UKCP09) downscaled from the Met Office regional climate model were implemented in both precipitation and evapotranspiration timeseries. The highest changes in both the annual mean precipitation and potential evapotranspiration, considering a 90% probability level under a high-emission future scenario, projected for the 2080s relative to the baseline period of 1961–1990, were considered and distributed to the daily time scale (see Murphy et al. 2009 and Thompson, 2012). To include the probabilistic component in the hazard assessment, the precipitation dataset was manually modified implementing several events with 2, 5, 10, 30, 50, 100 and 500-year future return periods. For this purpose, the 24-hour cumulated rainfall Intensity Duration Frequency curves for the City of London under changing climatic conditions were used (see Prodanovic and Simonovic, 2007). | -Thompson, 2012  -Coxon et al. 2020  -<https://nrfa.ceh.ac.uk/data/search> |
| WATER PERCOLATING PERCENTAGE DUE TO GROUND TYPE  *(mm/Day)* | Constant |  | 0.5 |  | -Jones et al. 2012  -Stàsko et al. 2012  -Brooks, 2013  -Butler et al. 2018 |
| percolating flow  *(mm/Day)* | Auxiliary | IF THEN ELSE(infiltrating flow>EVAPOTRANSPIRATION, (infiltrating flow-EVAPOTRANSPIRATION)*WATER PERCOLATING PERCENTAGE DUE TO GROUND TYPE , 0 ) |  |  | -Brooks, 2013 |
| groundwater level increase rate  *(mm/Day)* | Inflow | percolating flow |  |  |  |
| Groundwater Level  *(mm)* | Stock |  | 29760 | Considering EA (2009), station n.16/TQ48_72D was chosen to understand the groundwater level on day 1. The data were taken from EA. By utilizing the total aquifer height (derived from the table on page 7 of the PBA (2017) – 30 m) and the initial water level value for the first day of 2010 (-0.24), the water height in meters was determined. Subsequently, this value was converted into millimeters.  30000-240 =29760 mm | -Groundwater levels data from EA  -EA, 2009  -PBA, 2017 |
| LAG TIME OF CONTRIBUTION  *(Day/Dmnl)* | Constant |  | 12 | Only 12 days were considered due to the small area under investigation, which spans 10 km2. | -Winter et al. 1998 |
| FRACTION OF BASEFLOW FROM GROUNDWATER  *(Dmnl)* | Constant |  | 0.00013 | According to data from <https://nrfa.ceh.ac.uk/data/station/meanflow/39001> , 63% of the water in the tidal river originates from stored sources. However, considering the proportion of the river's discharge attributed to other sources, which amounts to 7% (calculated using the discharge duration curve), 63% is adjusted to 56% (63%-7%). Given that the recording period spans 137 years (from 1883-2020), totalling 50,005 days, and stored sources contribute to the river's flow every 12 days, we divide the adjusted percentage (0.56) by 4,167 days (corresponding to 50,005 divided by 12).  0.63-0.07=0.56  137*365=50005 days  50005/12=4167 days  (0.56/4167) = 0.00013 | -<https://nrfa.ceh.ac.uk/data/station/meanflow/39001>  -Gustard et al. 1992  -Kelly et al. 2019 |
| groundwater contribution to tidal river  *(mm/Day)* | Auxiliary | (FRACTION OF BASEFLOW FROM GROUNDWATER*Tidal River Water Level)/LAG TIME OF CONTRIBUTIO**N** |  |  | -https://pubs.usgs.gov/circ/circ1186/html/gen_facts.html  -https://www.usgs.gov/special-topics/water-science-school/science/rivers-contain-groundwater  -http://www.columbia.edu/~vjd1/streams_basic.htm  -Kelly et al. 2019 |
| groundwater level decrease rate  *(mm/Day)* | Ouflow | groundwater contribution to tidal river |  |  |  |
| surface runoff due to soil moisture conditions  *(mm/Day)* | Auxiliary | IF THEN ELSE(Groundwater Level>=GROUNDWATER THRESHOLD, percolating flow , 0 ) |  |  |  |
| not percolating flow  *(mm/Day)* |  | IF THEN ELSE(infiltrating flow>EVAPOTRANSPIRATION, infiltrating flow-EVAPOTRANSPIRATION-percolating flow , 0 ) |  |  |  |
|  |  | **PLUVIAL FLOOD SECTION** |  |  |  |
| surface runoff  *(mm/Day)* | Auxiliary | precipitation's impervious component+not percolating flow+surface runoff due to soil moisture conditions+tidal river flood depth |  |  |  |
| FRACTION OF SURFACE RUNOFF AVAILABLE FOR SURFACE SYSTEM  *(Dmnl)* | Constant |  | 0.474 | By using Google My Maps the total area of the sub-catchments labelled "combined" and "storm" in Figure 2-4, JBA (2020)* were calculated. Subsequently, using a proportional approach, we determined the percentage of runoff directly contributing to the sewage system.  *Please note that the sub-catchment labelled "foul" was excluded from consideration as it does not pertain to surface runoff.  Total area: 10.2 km²  Area of sub-catchments:  "Combined": 2.21 km²  "Storm": 1.05 km² + 0.571 km² + 1.01 km² = 2.631 km²  Total area of "Combined" and "Storm" sub-catchments: 2.21 km² + 2.631 km² = 4.841 km²  To find the percentage of runoff directly to the sewage system:  10.2 km² : 100% = 4.841 km² : x  x ≈ 47.4% = 0.474 (runoff to sewage system | -JBA, 2020 |
| surface runoff to stormwater system  *(mm/Day)* | Auxiliary | surface runoff*FRACTION OF SURFACE RUNOFF AVAILABLE FOR SEWERAGE SYSTEM |  |  |  |
| STORMWATER SYSTEM AVAILABILITY AFFECTED BY AGEING/BLOCKAGE  *(mm/Day)* | Lookup | [(0,0)-(22266,200)],(1,5),(3,104),(179,4.9), (181,103),(729,4.8),(731,97),(879,4.7), (881,95),(1094,4.6),(1096,94),(1244,4.5), (1246,92),(1460,4.4),(1462,90),(1609,4.3), (1701,89),(1824,4.2),(1826,86),(1974,4.1), (1976,85),(2189,4),(2191,83),(2339,3.9), (2341,81),(2554,3.8),(2556,79),(2704,3.7), (2706,78),(2919,3.6),(2921,76),(3069,3.5), (3071,74),(3284,3.4),(3286,72),(3434,3.3), (3436,71),(3649,3.2),(3651,68),(3799,3.1), (3801,67),(4014,3),(4016,65),(4164,2.9), (4166,63),(4379,2.8),(4381,61),(4529,2.7), (4531,60),(4744,2.6),(4746,58),(4894,2.5), (4896,56),(5110,2.4),(5112,54),(5259,2.3), (5261,53),(5474,2.1),(5476,50),(5839,2), (5841,47),(5989,1.9),(5991,45),(6204,1.8), (6206,43),(6354,1.7),(6356,41),(6569,1.6), (6571,39),(6719,1.5),(6721,37),(6935,1.4), (6937,35),(7084,1.3),(7086,33),(7299,1.2), (7301,31),(7449,1.1),(7451,29),(7664,1), (7666,27),(7814,0.9),(7816,25),(8029,0.8), (8031,23),(8179,0.7),(8181,21),(8394,0.6), (8396,19),(8544,0.5),(8546,17),(8760,0), (8762,15),(8909,0),(8911,13),(9124,0), (9126,11),(9274,0),(9276,9),(9489,0), (9491,7),(9639,0),(9641,5),(9854,0),(9856,3),(10004,0),(10006,1),(10219,0),(10221,0), (10950,0),(12775,0),(14600,0),(16425,0), (18250,0),(20075,0),(21900,0),(23725,0), (25550,0),(27375,0) |  | Taking into account the typical lifespan of a sewerage system, which is approximately 40 years, and acknowledging maintenance work carried out on the Crossness sewage system in 2010, we ascertain that the storage capacity reached its maximum level (144 mm) in 2010, gradually diminishing to zero by 2050. The storage capacity for 2010 was determined based on insights from an expert, who stated: 'In general, sewer designers must adhere to regulations. If I recall correctly, the regulation specifies that a sewer should not flood when rainfall intensity reaches 6 mm/h, but it can tolerate flooding at higher intensities. By this logic, a sewer can store up to 6 * 24 mm = 0.144 m of rain per day, assuming rainfall is evenly distributed throughout the day.'  For subsequent years, the storage level decreases by 18 mm every 5 years (144:40 years = x:5 years).  To evaluate the frequency of sewer blockages, past blockage events were examined, resulting in residual system availability ranging from 0.5 mm to 15 mm effectiveness (without blockage) and 0 mm below 15 mm effectiveness. A blockage rate was derived from 2018 onwards, noting an increase in blockage events in that year. Therefore, from 2018 to 2050 (the 40-year lifespan of the system), it was assumed that there would be two blockages per year, occurring every 6 months (in January and June, as these months historically experienced the highest frequency of blockages). It is presumed that the system will be unblocked within 2 days each time it becomes blocked, consistent with past practices.  Effective from 2050 onwards, an effectiveness rating of 0 was considered due to the system's ageing state, necessitating new interventions. | -JBA, 2020  -https://www.water-technology.net/projects/crossness-sewage-treatment-works-upgrade/Thamesmead Bexley Flooding Database.xls |
| stormwater system capacity  *(mm/Day)* | Auxiliary | STORMWATER SYSTEM AVAILABILITY AFFECTED BY AGEING/BLOCKAGE (Time) |  |  |  |
| pluvial flood depth  *(mm/Day)* | Auxiliary with Lookup | surface runoff to stormwater system-stormwater system capacity    ([(-100,0)-(100,100)],(-70,0),(-60,0),(-50,0), (-40,0),(-30,0),(-20,0),(-10,0), (1,0),(0,0),(1,1),(10,10),(20,20),(30,30), (40,40),(50,50),(60,60),(70,70) ) |  |  |  |
| stormwater to lakes and canals  *(mm/Day)* | Auxiliary | IF THEN ELSE(pluvial flood depth=0, surface runoff to stormwater system , surface runoff to stormwater system-pluvial flood depth ) |  |  |  |
| DELAY IN STORMWATER DISCHARGE INTO LAKES AND CANALS  *(Day)* | Constant |  | 1 |  |  |
| delayed stormwater discharge into lakes and canals  *(mm/Day)* | Auxiliary | DELAY1( stormwater to lakes and canals, DELAY IN STORMWATER DISCHARGE INTO LAKES AND CANALS ) |  |  |  |
| pluvial flood hazard index  *(Dmnl)* | Auxiliary with Lookup | surface runoff to stormwater system-stormwater system capacity    ([(0,0)-(19,10)],(-60,1), (-10,1.5),(0,1.5),(0.72,2),(4.32,2),(5.04,2), (14.4,2),(15.12,2.5),(16.2,2.5),(16.56,3),(18,3),(18.72,3) ) |  |  | -Tingsanchali and Promping, 2022 |
|  |  | **FLUVIAL FLOOD SECTION** |  |  | · |
| FRACTION OF SURFACE RUNOFF AVAILABLE FOR LAKES AND CANALS  *(Dmnl)* | Constant |  | 0.526 | By using Google My Maps the total area of the sub-catchments labelled "combined" and "storm" in Figure 2-4, JBA (2020)* were calculated. Subsequently, using a proportional approach, we determined the percentage of runoff directly contributing to the sewage system. Then, we calculated that 52.6% of runoff is directed to the drainage system.  *Please note that the sub-catchment labelled "foul" was excluded from consideration as it does not pertain to surface runoff.  Total area: 10.2 km²  Area of sub-catchments:  "Combined": 2.21 km²  "Storm": 1.05 km² + 0.571 km² + 1.01 km² = 2.631 km²  Total area of "Combined" and "Storm" sub-catchments: 2.21 km² + 2.631 km² = 4.841 km²  To find the percentage of runoff directly to the sewage system:  10.2 km² : 100% = 4.841 km² : x  x ≈ 47.4% = 0.474 (runoff to sewage system  1-0.474= 0.526 (runoff to drainage system) | -JBA, 2020 |
| surface runoff to lakes and canals  *(mm/Day)* | Auxiliary | (surface runoff*FRACTION OF SURFACE RUNOFF AVAILABLE FOR LAKES AND CANALS)+pluvial flood depth+delayed stormwater discharge into lakes and canals |  |  |  |
| THAMESMEAD AREA  *(sqm)* | Constant |  | 10.2 |  | -Peabody, 2021 |
| LAKES AREA  *(sqm)* | Constant |  | 7 |  | -JBA, 2020 |
| water in lakes  *(mm/Day)* | Auxiliary | (LAKES AREA*surface runoff to lakes and canals)/THAMESMEAD AREA |  |  |  |
| water in SUDS increase rate  *(mm/Day)* | Inflow | water in lakes |  |  |  |
| Water in SUDS  *(mm)* | Stock | water in SUDS increase rate-water in SUDS decrease rate | 0 |  | -Peabody, 2021  -GLC Paper on Surface Water Drainage  -URS Scott Wilson, 2012 |
| water in canals  *(mm/Day)* | Auxiliary | surface runoff to lakes and canals |  |  |  |
| CANALS AVAILABILITY AFFECTED BY AGEING/BLOCKAGE  *(mm/Day)* | Lookup | [(0,0)-(22266,20000)],(1,760),(1460,685), (3285,548),(5110,411),(6935,274),(8760,137),(10950,0),(12775,0),(14600,0),(16425,0), (18250,0),(20075,0),(21900,0),(23725,0), (25550,0),(27375,0) |  | Canals have a standing water depth of 0.61m (610 mm) and a storage depth for accommodating additional stormwater of 0.76m (760 mm) from mean sea level. Tot 1370 mm. Considering that they were constructed in 1960 and that the last de-siltation took place 20 years ago (2000), a 10% reduction every 5 years in their availability (due to sediment build-up and ageing) was considered. Considering that surface runoff influences the additional stormwater depth (and not the standing one), the reduction in canals availability was considered only when it fell below the additional depth. This meant that from 2010 to 2020 the availability was equal to the additional depth. In fact, calculating a 10% reduction of 1370 mm each year, until 2020 the availability was more than 760 mm.  2000(year), 1370(availability)  2005, 1370-10%*1370=1233  2010, 1370-20%*1370=1096  2015, 959 (30%)  2020, 822 (40%)  From 2050 onwards an effectiveness of 0 was considered because the system will be old and will need also another de-siltation. | -JBA, 2020  -Peabody, 2021  -GLC Paper on Surface Water Drainage  -URS Scott Wilson, 2012 |
| canals capacity  *(mm/Day)* | Auxiliary | CANALS AVAILABILITY AFFECTED BY AGEING/BLOCKAGE (Time) |  |  |  |
| canals flood depth  *(mm/Day)* | Auxiliary with Lookup | canals capacity-water in canals    ([(-995,0)-(100,1000)],(-995,995), (-650,650),(-595,595),(-400,400),(-300,300), (-250,250),(0,0),(100,0) ) |  |  |  |
| canals flood hazard index  *(Dmnl)* | Auxiliary with Lookup | water in canals-canals capacity    ([(0,0)-(99,10)],(-300,1),(-200,1), (-100,1.5),(0,1.5),(3.8,2),(22.8,2),(26.6,2), (76,2),(79.8,2.5),(85.5,2.5),(87.4,3),(95,3), (98.8,3) ) |  |  | -Tingsanchali and Promping, 2022 |
| PUMPS AVAILABILITY AFFECTED BY AGEING/BLOCKAGE  *(mm/Day)* | Lookup | [(0,0)-(22266,5000)],(1,4450),(1460,4005), (3285,3560),(5110,3115),(6935,2670), (8760,2225),(10950,1780),(12775,1335), (14600,890),(16425,445),(18250,0),(20075,0),(21900,0),(23725,0),(25550,0),(27375,0) |  | The maximum availability of pumps was calculated by summing the differences between mm of switch on and switch off of each pump.  Then, to consider the build-up of pumps, a reduction of 10% every 5 years in the availability of pumps was considered.  *Great Brach (0.15+0.13+0.2) = 0.48m  Green Level (0.75+0.95+1.15) = 2.85m  Lake5 (0.15+0.15+0.025+0.075) = 0.4m  Lake4 (0.09+0.21+0.21+0.21) = 0.72m  0.48+2.85+0.4+0.72=4.450m =4450mm  2000(year), 4450  2005, 4450-10%*4450=4005  From 2050 onwards an availability equal to 0 was considered because the system will be old and will need also a de-siltation. | -JBA, 2020 |
| pumps capacity  *(mm/Day)* | Auxiliary | PUMPS AVAILABILITY AFFECTED BY AGEING/BLOCKAGE (Time) |  |  |  |
| THRESHOLD FOR PUMPS ACTIVATION  *(mm)* | Constant |  | 150 |  | -GLC Paper on Surface Water Drainage |
| water to pumping stations  *(mm/Day)* | Auxiliary | IF THEN ELSE(Water in SUDS>=THRESHOLD FOR PUMPS ACTIVATION, Water in SUDS/DAY , 0 ) |  |  |  |
| pumped water  *(mm/Day)* | Auxiliary | IF THEN ELSE(water to pumping stations<=pumps capacity, water to pumping stations , pumps capacity ) |  |  |  |
| SUDS THRESHOLD  *(mm/Day)* | Constant |  | 760 | SUDS have a standing water depth of 1.22m (1220 mm) and a storage depth for accommodating additional storm water of 0.76m (760 mm) from mean sea level. | -GLC Paper on Surface Water Drainage |
| SUDS flood depth  *(mm/Day)* | Auxiliary | IF THEN ELSE(water to pumping stations-pumped water>SUDS THRESHOLD, (water to pumping stations-pumped water-SUDS THRESHOLD), 0 ) |  |  |  |
| SUDS flood hazard index  *(Dmnl)* | Auxiliary with Lookup | (water to pumping stations-pumped water)-SUDS THRESHOLD    ([(0,0)-(99,10)],(-300,1),(-200,1), (-100,1.5),(0,1.5),(3.8,2),(22.8,2),(26.6,2), (76,2),(79.8,2.5),(85.5,2.5),(87.4,3),(95,3), (98.8,3) ) |  |  | -Tingsanchali and Promping, 2022 |
| fluvial flood hazard index  (Dmnl) | Auxiliary | MAX(canals flood hazard index,SUDS flood hazard index) |  |  |  |
|  |  | **TIDAL RIVER FLOOD SECTION** |  |  |  |
| SEA LEVEL RISE  *(mm/Day)* | Lookup | [(0,0)-(22266,10)],  (1,0.01),(1825,0.01),(3650,0.01),  (5475,0.023),(7300,0.023),(9125,0.023),  (10950,0.023),(12775,0.023),(14965,0.023),  (16790,0.023),(18615,0.03),(20440,0.03),  (22266,0.03) |  |  | -EA, 2010 |
| sea level increase rate  *(mm/Day)* | Auxiliary | SEA LEVEL RISE (Time) |  |  |  |
| tidal river increase rate  *(mm/Day)* | Inflow | sea level increase rate |  |  |  |
| tidal river inflow  *(mm/Day)* | Inflow | delayed pumped water discharge into  river+overland flow |  |  |  |
| tidal river outflow  *(mm/Day)* | Outflow | DELAY1(delayed pumped water discharge into river+overland flow, 1) |  |  |  |
| Tidal River Water Level  *(mm)* | Stock | tidal river increase rate+tidal river inflow-tidal river outflow | 15000 |  | -EA, 2010  -EA, 2012 |
| TIDAL RIVER DEFENCES THRESHOLD AFFECTED BY AGEING  *(mm)* | Lookup | [(0,0)-(22266,30000)], (1,18600),(1460,18400), (3285,18200),(5110,18000),(6935,17800), (8760,17600),(10950,17400),(12775,17200), (14600,17000),(16425,16800),(18250,16600),(20075,16400),(21900,16200), (23725,16000),(25550,15800),(27375,15600) |  |  | -https://www.gov.uk/government/publications/thames-estuary-2100-te2100/thames-estuary-2100-key-findings-from-the-monitoring-review  -https://www.ice.org.uk/what-is-civil-engineering/what-do-civil-engineers-do/thames-barrier#:~:text=Construction%20began%20in%201974.,by%20the%20Queen%20in%201984.&text=The%20Thames%20Barrier%20is%20the,defence%20barrier%20in%20the%20world.  -http://www.floodsite.net/html/cd_task17-19/thamesmead_embayment.html  -https://www.constructex.co.uk/thamesmead-flood-wall  -AECOM, 2017  -EA, 2012  -Peabody, 2021 |
| tidal river defences effectiveness  *(mm)* | Auxiliary | TIDAL RIVER DEFENCES THRESHOLD AFFECTED BY AGEING (Time) |  |  |  |
| tidal river flood depth  *(mm)* | Auxiliary | tidal river defences effectiveness-Tidal River Water Level    ([(-8000,0)-(7000,8000)],(-5000,5000), (-4000,4000),(-3000,3000),(-2000,2000), (-1000,1000),(0,0),(1000,0),(2000,0),(3000,0), (4000,0),(5000,0) ) |  |  |  |
| tidal river flood hazard index  *(Dmnl)* | Auxiliary with Lookup | Tidal River Water Level-tidal river defences effectiveness    ([(-3000,0)-(3000,10)],(-3000,1),(-2000,1), (-1000,1.5),(-500,1.5), (0,1.5),(200,2),(700,2),(600,2),(2100,2.5), (2250,2.5),(2300,3),(2500,3),(2600,3) ) |  |  | -Tingsanchali and Promping, 2022 |
|  |  | **FLOOD HAZARD SUB-MODEL** |  |  |  |
| PLUVIAL FLOOD WEIGHT  *(Dmnl)* | Constant |  | 0.3 |  |  |
| weighted pluvial flood hazard index  *(Dmnl)* | Auxiliary | pluvial flood hazard index*PLUVIAL FLOOD WEIGHT |  |  |  |
| TIDAL RIVER FLOOD DEPTH  *(Dmnl)* | Constant |  | 0.4 |  |  |
| weighted tidal river flood hazard index  *(Dmnl)* | Auxiliary | TIDAL RIVER FLOOD WEIGHT*tidal river flood hazard index |  |  |  |
| FLUVIAL FLOOD WEIGHT  *(Dmnl)* | Constant |  | 0.3 |  |  |
| weighted fluvial flood hazard index  *(Dmnl)* | Auxiliary | FLUVIAL FLOOD WEIGHT*fluvial flood hazard index |  |  |  |
| flood hazard index  *(Dmnl)* | Auxiliary | weighted pluvial flood hazard index+weighted fluvial flood hazard index+weighted tidal river flood hazard index |  |  | -Tingsanchali and Promping, 2022 |
|  |  | **TANGIBLE DAMAGE EVALUATION**  **SUB-MODEL** |  |  |  |
| buildings damage due to pluvial flooding  *(euro/sqm)* | Auxiliary with Lookup | pluvial flood depth    ([(0,0)-(6000,2000)], (0,0),(500,716.04),(1000,859),(1500,931), (2000,1002),(2500,1074),(3000,1217) ) |  |  | -Penning-Rowsell et al. 2010Penning-Rowsell et al. 2010 |
| buildings damage due to fluvial flooding  *(euro/sqm)* | Auxiliary with Lookup | fluvial flood depth    ([(0,0)-(6000,2000)], (0,0),(500,716.04),(1000,859),(1500,931), (2000,1002),(2500,1074),(3000,1217)) |  |  | -Penning-Rowsell et al. 2010Penning-Rowsell et al. 2010 |
| buildings damage due to tidal river flooding  *(euro/sqm)* | Auxiliary with Lookup | tidal river flood depth    ([(0,0)-(6000,2000)], (0,0),(500,716.04),(1000,859),(1500,931), (2000,1002),(2500,1074),(3000,1217)) |  |  | -Penning-Rowsell et al. 2010 |
| buildings damage due to flooding  *(euro/sqm)* | Auxiliary | buildings damage due to pluvial flooding+ buildings damage due to fluvial flooding  +buildings damage due to tidal river flooding |  |  |  |
| buildings damage due to preparedness  *(euro/sqm)* | Auxiliary | SIMULTANEOUS (buildings damage due to flooding-(effect of households' preparedness on damage*buildings damage due to flooding),1) |  |  |  |
| residential buildings damage class  *(Dmnl)* | Auxiliary with Lookup | buildings damage due to preparedness    ([(0,0)-(11000,200)], (0,1),(108,1),(135,2),(271,2),(298,3) ) |  |  | -Tingsanchali and Promping, 2022 |
| businesses damage due to pluvial flooding  *(euro/sqm)* | Auxiliary with Lookup | pluvial flood depth    ([(0,0)-(6000,2000)], (0,0),(500,572),(1000,1073),(1500,1359), (2000,1573),(3000,1717)) |  |  | -Penning-Rowsell et al. 2010 |
| business damage due to fluvial flooding  *(euro/sqm)* | Auxiliary with Lookup | fluvial flood depth    ([(0,0)-(6000,2000)], (0,0),(500,572),(1000,1073),(1500,1359), (2000,1573),(3000,1717)) |  |  | -Penning-Rowsell et al. 2010 |
| business damage due to tidal river flooding  *(euro/sqm)* | Auxiliary with Lookup | tidal river flood depth    ([(0,0)-(6000,2000)], (0,0),(500,572),(1000,1073),(1500,1359), (2000,1573),(3000,1717)) |  |  | -Penning-Rowsell et al. 2010 |
| business damage due to flooding  *(euro/sqm)* | Auxiliary | businesses damage due to pluvial flooding+businesses damage due to fluvial flooding  +businesses damage due to tidal river flooding |  |  |  |
| business damage class  *(Dmnl)* | Auxiliary with Lookup | business damage due to flooding    ([(0,0)-(11000,200)], (0,1),(108,1),(135,2),(271,2),(298,3) ) |  |  | -Tingsanchali and Promping, 2022 |
| recreational facilities damage due to pluvial flooding  *(euro/sqm)* | Auxiliary with Lookup | pluvial flood depth    ([(0,0)-(3000,2000)], (0,0),(500,437),(1000,728),(1500,1020), (2000,1093),(2500,1166),(3000,1239) ) |  |  | -Penning-Rowsell et al. 2010 |
| recreational facilities damage due to fluvial flooding  *(euro/sqm)* | Auxiliary with Lookup | fluvial flood depth    ([(0,0)-(3000,2000)], (0,0),(500,437),(1000,728),(1500,1020), (2000,1093),(2500,1166),(3000,1239) ) |  |  | -Penning-Rowsell et al. 2010 |
| recreational facilities damage due to tidal river flooding  *(euro/sqm)* | Auxiliary with Lookup | tidal river flood depth    ([(0,0)-(3000,2000)], (0,0),(500,437),(1000,728),(1500,1020), (2000,1093),(2500,1166),(3000,1239) |  |  | -Penning-Rowsell et al. 2010 |
| recreational facilities damage due to flooding  *(euro/sqm)* | Auxiliary | recreational facilities damage due to pluvial flooding+recreational facilities damage due to fluvial flooding  +recreational facilities damage due to tidal river flooding |  |  |  |
| recreational facilities damage class  *(Dmnl)* | Auxiliary with Lookup | recreational facilities damage due to flooding    ([(0,0)-(22,10)],(12,1),(13,2),(20,2),(22,3) ) |  |  | -Tingsanchali and Promping, 2022 |
| transport services damage due to pluvial flooding  *(euro/sqm)* | Auxiliary with Lookup | pluvial flood depth    ([(0,0)-(3000,900)], (0,0),(500,291),(1000,437),(1500,583), (2000,655),(2500,728),(3000,801) ) |  |  | -Penning-Rowsell et al. 2010 |
| transport services damage due to fluvial flooding  *(euro/sqm)* | Auxiliary with Lookup | fluvial flood depth    ([(0,0)-(3000,900)], (0,0),(500,291),(1000,437),(1500,583), (2000,655),(2500,728),(3000,801) ) |  |  | -Penning-Rowsell et al. 2010Penning-Rowsell et al. 2010 |
| transport services damage due to tidal river flooding  *(euro/sqm)* | Auxiliary with Lookup | tidal river flood depth    ([(0,0)-(3000,900)], (0,0),(500,291),(1000,437),(1500,583), (2000,655),(2500,728),(3000,801) ) |  |  | -Penning-Rowsell et al. 2010Penning-Rowsell et al. 2010 |
| transport services damage due to flooding  *(euro/sqm)* | Auxiliary | transport services damage due to pluvial flooding+transport services damage due to fluvial flooding  +transport services damage due to tidal river flooding |  |  |  |
| transport services damage class  (Dmnl) | Auxiliary with Lookup | transport services damage due to flooding    ([(0,0)-(20,10)],(12,1),(13,2),(14,3),(20,2) ) |  |  | -Tingsanchali and Promping, 2022 |
| tangible damage class due to flooding  *(Dmnl)* | Auxiliary | SIMULTANEOUS(MAX(businesses damage class, MAX(recreational facilities damage class, MAX(residential buildings damage class, transport services damage class  ))),1) |  |  |  |
|  |  | **ECOSYSTEM QUALITY SECTION** |  |  |  |
| effect of damage on transport services  *(Dmnl)* | Auxiliary with Lookup | transport services damage class    ([(0,0)-(10,10)],(1,0),(2,0.5),(3,1) ) |  |  |  |
| TRANSPORT SERVICES  *(Dmnl)* | Lookup | [(0,0)-(22266,10)], (1,0.1),(1460,0.1),(3285,0.1),(5110,0.1), (6935,0.5),(8760,0.5),(10950,0.7),(12775,0.7),(14600,0.7),(16425,0.7),(18250,0.7), (20075,0.7),(21900,0.7),(23725,0.7), (25550,0.7),(27375,0.7) |  |  | -O’Keeffe et al. 2022 |
| transport services over time  *(Dmnl)* | Auxiliary | TRANSPORT SERVICES (Time) |  |  |  |
| transport services due to damage  *(Dmnl)* | Auxiliary | transport services over time-(effect of damage on transport services*transport services over time) |  |  |  |
| effect of damage on facilities availability  *(Dmnl)* | Auxiliary with Lookup | recreational facilities damage class    ([(0,0)-(10,10)],(1,0),(2,0.5),(3,1) ) |  |  |  |
| AVAILABILITY OF FACILITIES  *(Dmnl)* | Constant |  | 0.1 |  | -O’Keeffe et al. 2022 |
| availability of facilities due to damage  *(Dmnl)* | Auxiliary | AVAILABILITY OF FACILITIES-(AVAILABILITY OF FACILITIES*effect of damage on facilities availability) |  |  |  |
| PROXIMITY TO NATURAL SPACES  *(Dmnl)* | Constant |  | 0.5 |  | -O’Keeffe et al. 2022 |
| NUMBER OF VARIABLES ON GREEN SPACES EXPERIENCE  *(Dmnl)* | Constant |  | 4 |  |  |
| green spaces experience  *(Dmnl)* | Auxiliary | (Ecosystem Quality State+PROXIMITY TO NATURAL SPACES+availability of facilities due to damage+transport services due to damage  )/NUMBER OF VABIALES ON GREEN SPACES EXPERIENCE |  |  | -O’Keeffe et al. 2022 |
| Ecosystem Quality State  *(Dmnl)* | Stock | ecosystem quality state increase rate-ecosystem quality state decrease rate | 0.3 |  | -Stakeholders’ BOT graphs (Workshop n.1) |
| effect of low flood depth on ecosystem quality  *(Dmnl)* | Auxiliary with Lookup | flood hazard index    ([(0,0)-(10,10)],(2,0.5),(2.5,0),(3,0) ) |  |  | -Maher et al.2014 |
| ecosystem quality state increase rate  *(Dmnl/Day)* | Inflow | Ecosystem Quality State*effect of low flood depth on ecosystem quality/DAYS |  |  |  |
| effect of high flood depth on ecosystem quality  *(Dmnl)* | Auxiliary with Lookup | flood hazard index    ([(0,0)-(10,10)],(2,0),  (2.5,0),(3,0.53) ) |  |  | -Talbot et al. 2018  -Zhang et al. 2021  -Peabody, 2021 |
| effect of imperviousness on ecosystem quality  *(Dmnl)* | Auxiliary with Lookup | Imperviousness coefficient    ([(0,0)-(10,10)], (0.2,0),(0.4,0),(0.6,0.25),(0.8,0.5),(1,0.75) ) |  |  | -Yan et al. 2019  -O’Keeffe et al. 2022 |
| ecosystem quality state decrease rate  *(Dmnl/Day)* | Outflow | (Ecosystem Quality State*effect of imperviousness on ecosystem quality/DAYS)+(Ecosystem Quality State  *effect of high flood depth on ecosystem quality  /DAYS)+(Ecosystem Quality State*green spaces experience/DAYS) |  |  |  |
|  |  | **COMMUNITY FLOOD RISK PERCEPTION SECTION** |  |  |  |
| INFORMATION SYSTEMS EFFECTIVENESS CLASS  *(Dmnl)* | Constant |  | 1 |  | -Stakeholders’ individual interviews |
| INFORMATION SYSTEMS EFFECTIVENESS WEIGHT IN PERCEPTION  *(Dmnl)* | Constant |  | 0.1 |  |  |
| information systems effectiveness class in perception  *(Dmnl)* | Auxiliary | INFORMATION SYSTEMS EFFECTIVENESS CLASS*INFORMATION SYSTEMS EFFECTIVENESS WEIGHT IN PERCEPTION |  |  |  |
| CITIZENS' INVOLVEMENT CLASS  *(Dmnl)* | Constant |  | 1 |  | -Stakeholders’ individual interviews |
| DELAY IN CITIZENS' INVOLVEMENT  *(Day)* | Constant |  | 365 |  |  |
| CITIZENS' INVOLVEMENT WEIGHT  *(Dmnl)* | Constant |  | 0.35 |  |  |
| local community engagement class  *(Dmnl)* | Auxiliary | DELAY1(CITIZENS' INVOLVEMENT CLASS*CITIZENS' INVOLVEMENT WEIGHT, DELAY IN CITIZENS' INVOLVEMENT) |  |  |  |
| FLOOD MONITORING AND WARNING SYSTEMS EFFECTIVENESS CLASS  *(Dmnl)* | Constant |  | 2 |  | -Stakeholders’ individual interviews |
| FLOOD MONITORING AND WARNING SYSTEMS EFFECTIVENESS WEIGHT IN PERCEPTION  *(Dmnl)* | Constant |  | 0.1 |  |  |
| monitoring and warning systems effectiveness class in perception  *(Dmnl)* | Auxiliary | FLOOD MONITORING AND WARNING SYSTEMS EFFECTIVENESS CLASS*FLOOD MONITORING AND WARNING SYSTEMS EFFECTIVENESS WEIGHT IN PERCEPTION |  |  |  |
| DAMAGE DUE TO FLOODING WEIGHT IN PERCEPTION  *(Dmnl)* | Constant |  | 0.35 |  |  |
| COMMUNITY SENSE OF SAFETY CLAS  *(Dmnl)* | Constant |  | 1 |  | -Stakeholders’ individual interviews |
| COMMUNITY SENSE OF SAFETY WEIGHT IN PERCEPTION  *(Dmnl)* | Constant |  | 0.1 |  |  |
| community sense of safety class in perception  *(Dmnl)* | Auxiliary | COMMUNITY SENSE OF SAFETY CLASS*COMMUNITY SENSE OF SAFETY WEIGHT IN PERCEPTION |  |  |  |
| Community Flood Risk Perception  *(Dmnl)* | Stock | community perception increase rate-community perception decrease rate | 1 |  | -Bradford et al. 2012  -Lechowska, 2018 |
| community perception increase rate  *(Dmnl/Day)* | Inflow | (damage class due to flooding in perception+local community engagement class+monitoring and warning systems effectiveness class in perception+information systems effectiveness class in perception  )/DAYS |  |  |  |
| community perception decrease rate  *(Dmnl/Day)* | Outflow | community sense of safety class in perception/DAYS |  |  |  |
| COMMUNITY FLOOD RISK PERCEPTION WEIGHT IN PREPAREDNESS  *(Dmnl)* | Constant |  | 0.6 |  | -Cologna et al. 2017  -Papagiannaki et al. 2019  -Liu et al. 2022 |
| community flood risk perception in preparedness  *(Dmnl)* | Auxiliary | Community Flood Risk Perception*COMMUNITY FLOOD RISK PERCEPTION WEIGHT IN PREPAREDNESS |  |  |  |
| DAMAGE DUE TO FLOODING WEIGHT IN PREPAREDNESS  *(Dmnl)* | Constant |  | 0.3 |  | -Cologna et al. 2017  -Papagiannaki et al. 2019  -Liu et al. 2022 |
| COMMUNITY SENSE OF SAFETY WEIGHT IN PREPAREDNESS  *(Dmnl)* | Constant |  | 0.1 |  | -Cologna et al. 2017  -Papagiannaki et al. 2019  -Liu et al. 2022 |
| community sense of safety class in preparedness  *(Dmnl)* | Auxiliary | COMMUNITY SENSE OF SAFETY CLASS*COMMUNITY SENSE OF SAFETY WEIGHT IN PREPAREDNESS |  |  |  |
| class of preparedness towards flooding  *(Dmnl)* | Auxilary | SIMULTANEOUS(community flood risk perception in preparedness+community sense of safety class in preparedness+damage class due to flooding in preparedness,1) |  |  |  |
| effect of households' preparedness on damage  *(Dmnl)* | Auxiliary with Lookup | class of preparedness towards flooding    ([(0,0)-(10,10)], (1,0),(1.5,0.075),(2,0.15),(2.5,0.225),(3,0.3) ) |  |  | -Messner and Meyer, 2006 |
|  |  | **RESIDENTS’ WELL-BEING SECTION** |  |  |  |
| effect of ecosystem quality on residents' well-being  *(Dmnl)* | Auxiliary | IF THEN ELSE(Ecosystem Quality State>ECOSYSTEM QUALITY STATE INITIAL VALUE, 0.15 , 0 ) |  |  | -Bratman et al. 2019  -Salvia et al. 2022 |
| effect of green spaces experience on well-being  *(Dmnl)* | Auxiliary with Lookup | green spaces experience    ([(0,0)-(10,10)],(0,0),(0.5,0.5),(1,1) ) |  |  | -Bratman et al. 2019  -Salvia et al. 2022 |
| effect of attractiveness on residents' well-being  *(Dmnl)* | Auxiliary with Lookup | Attractiveness for Companies    ([(0,0)-(10,10)],(0.1,0.15),(0.5,0.25),(1,0.5) ) |  |  |  |
| Residents’ Well-Being  *(Dmnl)* | Stock | well-being increase rate - well-being decrease rate | 0.25 |  | -Stakeholders’ BOT graphs (Workshop n.1) |
| well-being increase rate  *(Dmnl/Day)* | Inflow | IF THEN ELSE(Attractiveness for Companies>ATTRACTIVENESS FOR COMPANIES INITIAL VALUE, (effect of attractiveness on residents' well-being  *Residents' Well-Being  /DAYS)+(effect of green spaces experience on well-being  *Residents' Well-Being)/DAYS+(effect of ecosystem quality on residents' well-being*Residents' Well-Being)/DAYS, (effect of green spaces experience on well-being  *Residents' Well-Being)/DAYS)+(effect of ecosystem quality on residents' well-being*Residents' Well-Being)/DAYS |  |  | -Bratman et al. 2019 |
| effect of flood hazard on well-being  *(Dmnl)* | Auxiliary with Lookup | flood hazard index    ([(0,0)-(10,10)],(1,0),(2,0),(2.5, 0.17),(3,0.25) ) |  |  | -Foudi et al. 2017 |
| effect of flood perception on well-being  *(Dmnl)* | Auxiliary with Lookup | Community Flood Risk Perception    ([(0,0)-(10,10)],(1,0.29),(2,0.23),(3,0.18) ) |  |  | -Foudi et al. 2017 |
| effect of damage on well-being  *(Dmnl)* | Auxiliary with Lookup | tangible damage class due to flooding    ([(0,0)-(10,10)],(1,0.19),(2,0.25),(3,0.32) ) |  |  | -Foudi et al. 2017 |
| well-being decrease rate  *(Dmnl/Day)* |  | (Residents' Well-Being*effect of flood hazard on well-being/DAYS)+(Residents' Well-Being*effect of damage on well-being  /DAYS)+(Residents' Well-Being*effect of flood perception on well-being/DAYS) |  |  | -Foudi et al. 2017  -French et al. 2019  -Lee et al. 2020  -Robin et al. 2020 |
|  |  | **ATTRACTIVENESS FOR COMPANIES**  **SECTION** |  |  |  |
| effect of ecosystem quality on attractiveness  *(Dmnl)* | Auxiliary | IF THEN ELSE(Ecosystem Quality State>ECOSYSTEM QUALITY STATE INITIAL VALUE, 0.15 , 0 ) |  |  | -<https://www.wur.nl/en/show-longread/Seven-Reasons-to-Invest-in-a-Green-City.htm>  -The Land Trust, 2018 |
| effect of residents' well-being on attractiveness  *(Dmnl)* | Auxiliary | IF THEN ELSE("Residents' Well-Being">"RESIDENTS' WELL-BEING INITIAL VALUE", 0.15 , 0 ) |  |  | -Frumkin,2003  -Bond et al. 2012 |
| effect of damage on attractiveness  *(Dmnl)* | Auxiliary with Lookup | tangible damage class due to flooding    ([(0,0)-(10,10)],(1,0),(2,0.5),(3,1) ) |  |  | -Bond et al. 2012 |
| ATTRACTIVENESS FOR COMPANIES INITIAL VALUE  *(Dmnl)* | Constant |  | 0.5 |  | -Stakeholders’ BOT graphs (Workshop n.1) |
| Attractiveness for Companies  *(Dmnl)* |  | attractiveness for companies increase rate-attractiveness for companies decrease rate |  |  |  |
| attractiveness for companies increase rate  *(Dmnl/Day)* | Inflow | (effect of ecosystem quality on attractiveness*Attractiveness for Companies  /DAYS)+("effect of residents' well-being on attractiveness"*Attractiveness for Companies/DAYS) |  |  |  |
| attractiveness for companies decrease rate  *(Dmnl/Day)* | Outflow | effect of damage on attractiveness*Attractiveness for Companies/DAYS |  |  |  |
|  |  | **FLOOD EXPOSURE SECTION** |  |  |  |
| BUILDINGS MATERIAL CATEGORY  *(Dmnl)* | Lookup | [(0,0)-(22266,10)], (1,2),(1460,2),(3285,2),(5110,2),(6935,3), (8760,3),(10950,3),(12775,3),(14600,3), (16425,3),(18250,3),(20075,3),(21900,3), (23725,3),(25550,3),(27375,3) |  |  | -Hall and Madden, 2018  -Validation workshop with stakeholders (Workshop n.2) |
| buildings material class  *(Dmnl)* | Auxiliary | BUILDINGS MATERIAL CATEGORY (Time) |  |  |  |
| EARNINGS GROWTH RATE  *(Dmnl)* | Lookup | [(0,0)-(22266,10)], (1,0.008),(1460,0.008),(3285,0.008), (5110,0.008),(6935,0.008),(8760,0.008), (10950,0.008),(12775,0.008),(14600,0.008), (16425,0.008),(18250,0.008),(20075,0.008), (21900,0.008),(23725,0.008),(25550,0.008), (27375,0.008) |  | The analysis was based on the graph in Hall and Madden (2018), Page 11. In 2010, earnings were 250, increasing to 280 by 2020, marking a 30-point increase over 10 years. To extrapolate the daily increase over 60 years (2010-2070), we calculated the average yearly increase by dividing 30 by 10, resulting in 3. Then, to find the yearly increase over 60 years, we multiplied 3 by 60, yielding 180. Dividing this by the total number of days in 60 years (22,266), we obtained the daily growth rate of 0.008. It was assumed that the rate of increase in people's income will remain constant over time, unaffected by other factors. | -Hall and Madden, 2018 |
| HOUSE PRICES CHANGE RATE DUE TO REGENERATION AND POPULATION GROWTH  *(Dmnl)* | Lookup | [(0,0)-(14965,10)], (1,0.015),(1460,0.057),(3285,0.05), (5110,0.043),(6935,0.035),(8760,0.028), (10950,0.015), (12775,0.062), (14600,0.06), (16425,0.057),(18250,0.055),(20075,0.05), (21900,0.048),(23725,0.042),(25550,0.038), (27375,0.032) |  | The analysis is based on data from Hall and Madden (2018), Page 11. In 2010, house prices were at 300, rising to 350 by 2020, marking a 50-point increase over 10 years. To extrapolate this increase over 60 years (2010-2070), we calculated the average yearly increase by dividing 50 by 10, resulting in 5. Then, to find the yearly increase over 60 years, we multiplied 5 by 60, yielding 300. Dividing this by the total number of days in 60 years (22,266), we obtained the daily growth rate of 0.015.  The sharp increase in housing prices observed in new developments may be attributed to the high costs of regeneration, as mentioned in Hall and Madden (2018). This includes expenses such as demolition costs, construction costs, time-lag costs, home-loss payments, and rehousing costs. Additionally, population growth also influences housing demand and subsequently, prices. According to Peabody (2021), the population of Thamesmead is expected to increase from 40000 to 100000 by 2050, indicating a 2.5-fold increase.  Multiplying the house prices in 2020 (350) by this growth factor yields 875. Subtracting the initial price from this value (875 - 350) gives us 525. Dividing this by the number of days in 30 years (10,950, from 2020 to 2050), we find the daily house price growth due to population growth to be 0.05.  Housing supply is factored in starting from 2025, as indicated by Peabody (2021), which states that 24,000 new homes will be built by 2050. Assuming a price inelastic short-run supply of housing, we consider a 5-year lag for new construction to affect the market. Therefore, every 5 years starting from 2025, the growth rate of demand for houses (0.05/day) is met with a 15% increase in construction. This equates to a daily supply growth rate of 0.0075.  The difference between demand and supply (0.05 - 0.0075) gives us the change in price due to this factor. Adding this to the initial growth rate per regeneration (0.015) yields the overall change in price. This process is repeated for subsequent years, accounting for changes in both demand and supply.  It is anticipated that after 2050, demand will continue to outpace supply due to ongoing population growth, leading to further price increases. | -Miles,2012  -Hall and Madden, 2018  -Peabody,2021  -https://www.investopedia.com/ask/answers/040215/how-does-law-supply-and-demand-affect-housing-market.asp#:~:text=The%20housing%20market%20is%20a,less%20demand%20in%20the%20market.  -https://www.economicshelp.org/blog/377/housing/factors-that-affect-the-housing-market/  -https://pearsonblog.campaignserver.co.uk/supply-and-demand-the-housing-market/ |
| housing affordability  *(Dmnl)* | Auxiliary | HOUSE PRICES CHANGE RATE DUE TO REGENERATION AND POPULATION GROWTH(Time)/EARNINGS GROWTH RATE (Time) |  |  | -https://www.thesundaily.my/business/the-problem-of-measuring-housing-affordability-based-on-price-to-income-ratio-BC8730737 |
| housing affordability class  *(Dmnl)* | Auxiliary | housing affordability    ([(0,0)-(10,10)] ,(1,3),(2,3),(3,3),(3.1,2),(4,2),(4.1,1),(5,1), (6,1) ) |  |  | -https://www.thesundaily.my/business/the-problem-of-measuring-housing-affordability-based-on-price-to-income-ratio-BC8730737 |
| AVERAGE HOUSE AREA  *(sqm)* | Constant |  | 80 |  | -https://www.zoopla.co.uk/for-sale/property/thamesmead/  -https://www.designingbuildings.co.uk/wiki/Minimum_space_standards |
| space standard class  *(Dmnl)* | Auxiliary with Lookup | AVERAGE HOUSE AREA    ([(0,0)-(150,10)], (37,1),(49,1),(50,2),(99,2),(100,3),(150,3) ) |  |  | -https://www.designingbuildings.co.uk/wiki/Minimum_space_standards |
| CAR PARKING SPACE CLASS  *(Dmnl)* | Constant |  | 2 |  | -O’Keeffe et al.2022 |
| population density class  *(Dmnl)* | Auxiliary with Lookup | Density of Building Development    ([(0,0)-(100,10)], (5,1),(10,1),(20,2),(30,2),(40,3),(100,3) ) |  |  | -Landcom, 2011 |
| building quality class  *(Dmnl)* | Auxiliary | IF THEN ELSE(buildings material class=1:AND:space standard class=1:AND:population density class=3:AND:CAR PARKING SPACE CLASS  =1:AND:housing affordability class=1, 1 , buildings material class ) |  |  | -Nasiri et al. 2017 |
| SERVICES QUALITY CATEGORY  *(Dmnl)* | Constant | [(0,0)-(22266,10)], (1,1),(1460,1),(3285,1),(5110,1),(6935,2), (8760,2),(10950,2),  (12775,2),(14600,2), (16425,2),(18250,2),  (20075,2),(21900,2), (23725,2),(25550,2),  (27375,2) |  |  | -Peabody, 2021 |
| services quality class  *(Dmnl)* | Auxiliary | SERVICES QUALITY CATEGORY (Time) |  |  |  |
| INFRASTRUCTURE QUALITY CATEGORY  *(Dmnl)* | Lookup | [(0,0)-(22266,10)], (1,1),(1460,1),(3285,1),(5110,1),(6935,2), (8760,2),(10950,2),  (12775,2),(14600,2), (16425,2),(18250,2),  (20075,2),(21900,2), (23725,2),(25550,2),  (27375,2) |  |  | -Peabody, 2021 |
| infrastructure quality class  *(Dmnl)* | Auxiliary | INFRASTRUCTURE QUALITY CATEGORY (Time) |  |  |  |
| built environment quality class | Auxiliary | (buildings quality class+infrastructure quality class+services quality class)/3 |  |  |  |
| effect of built environment quality on flood exposure  *(Dmnl)* | Auxiliary with Lookup | built environment quality class    ([(0,0)-(10,10)],(1,3),  (2,2),(3,1) ) |  |  |  |
| BUILT ENVIRONMENT QUALITY WEIGHT  *(Dmnl)* | Constant |  | 0.4 |  |  |
| PROXIMITY TO FLOOD SOURCES CLASS  *(Dmnl)* | Constant |  | 2 | Google Maps was utilized to estimate the distance between flood sources (SUDS, sewage, Thames River) and properties. Drawing upon this data and considering the classification system outlined by Tingsanchali and Promping (2022) in Table 3, it was concluded that the case study falls within the medium class, denoted as class 2. This decision was made as the distances between flood sources and properties were found to be within 1500 meters at most, aligning with the criteria for the medium classification. | -Kissi et al. 2015  -Ntajal et al. 2016  -Tingsanchali and Promping, 2022  -Hamidi et al. 2022 |
| PROXIMITY TO FLOOD SOUCES WEIGHT  *(Dmnl)* | Constant |  | 0.4 |  |  |
| LAND USE TYPE CLASS  *(Dmnl)* | Constant |  | 2 |  | -Tingsanchali and Promping, 2022 |
| LAND USE TYPE WEIGHT  *(Dmnl)* | Constant |  | 0.2 |  |  |
| flood exposure factor  *(Dmnl)* | Auxiliary | (effect of built environment quality on flood exposure*BUILT ENVIRONMENT QUALITY WEIGHT)+(PROXIMITY TO FLOOD SOURCES CLASS*PROXIMITY TO FLOOD SOURCES WEIGHT)+(LAND USE TYPE CLASS*LAND USE TYPE WEIGHT) |  |  | -Kissi et al. 2015  -Ntajal et al. 2016  -Nasiri et al. 2017  -Babanawo et al. 2022  -Hamidi et al. 2022  -Tingsanchali and Promping, 2022 |
|  |  | **FLOOD SUSCEPTIBILITY SECTION** |  |  |  |
| FAMILIES SIZE  *(members)* | Constant |  | 3 |  | -<https://www.zoopla.co.uk/for-sale/property/thamesme>ad/ |
| FAMILIES SIZE WEIGHT  *(Dmnl)* | Constant |  | 0.15 |  |  |
| CRITICAL INFRASTRUCTURE PRESENCE CLASS  *(Dmnl)* | Constant |  | 2 |  | -EA,2012  -Salvia et al. 2022 |
| CRITICAL INFRASTRUCTURE PRESENCE WEIGHT  *(Dmnl)* | Constant |  | 0.15 |  |  |
| residents' well-being class in susceptibility  *(Dmnl)* | Auxiliary with Lookup | **Residents’ Well-Being**    **([(0,0)-(10,10)], (0,3),(0.3,3),(0.4,2),(0.5,2),(0.6,2),(0.7,1), (1,1) )** |  |  |  |
| RESIDENTS' WELL-BEING WEIGHT  *(Dmnl)* | Constant |  | 0.15 |  |  |
| community flood risk perception class in susceptibility  *(Dmnl)* | Auxiliary with Lookup | **Community Flood Risk Perception**    **([(0,0)-(10,10)],(1,3),(2,2),(3,1) )** |  |  |  |
| COMMUNITY FLOOD RISK PERCEPTION WEIGHT  *(Dmnl)* | Constant |  | 0.15 |  |  |
| ELDERLY AND CHILDREN CLASS  *(Dmnl)* | Constant |  | 1 |  | -Ntajal et al. 2016  -https://www.postcodearea.co.uk/postaltowns/london/se280hs/demographics/ |
| ELDERLY AND CHIL-DREN WEIGHT  (Dmnl) | Constant |  | 0.15 |  |  |
| flood susceptibility factor  *(Dmnl)* | Auxiliary | (community flood risk perception class in susceptibility*COMMUNITY FLOOD RISK PERCEPTION WEIGHT)+(CRITICAL INFRASTRUCTURE PRESENCE CLASS  *CRITICAL INFRASTRUCTURE PRESENCE WEIGHT)+(ELDERLY AND CHILDREN CLASS*ELDERLY AND CHILDREN WEIGHT)+(families size class*FAMILIES SIZE WEIGHT  )+(residents' well-being class in susceptibility*RESIDENTS' WELL-BEING WEIGHT) |  |  | -Kissi et al. 2015  -Ntajal et al. 2016  -Nasiri et al. 2017  -Babanawo et al. 2022  -Hamidi et al. 2022  -Tingsanchali and Promping, 2022 |
|  |  | **FLOOD ADAPTIVE CAPACITY SECTION** |  |  |  |
| delayed citizens' involvement class  *(Dmnl)* | Auxiliary | DELAY1(CITIZENS' INVOLVEMENT CLASS, DELAY IN CITIZENS' INVOLVEMENT ) |  |  |  |
| DRAINAGE SYSTEMS CHARACTERISTICS CLASS  *(Dmnl)* | Constant |  | 3 |  |  |
| DRAINAGE SYSTEMS CHARACTERISTICS WEIGHT  *(Dmnl)* |  |  | 0.1 |  |  |
| INSTITUTIONAL CAPACITY TO COPE WITH FLOODING  *(Dmnl)* | Lookup | [(0,0)-(27375,10)], (1,2),(1460,2),(3285,2),(5110,2),(6935,2), (8760,2),(10950,2),(12775,2),(14600,2), (16425,2),(18250,2),(20075,2),(21900,2), (23725,2),(25550,2),(27375,2) |  |  | -Peabody, 2021 |
| institutional capacity to cope with flooding over time  *(Dmnl)* | Auxiliary | INSTITUTIONAL CAPACITY TO COPE WITH FLOODING (Time) |  |  |  |
| HOUSEHOLDERS EDUCATION CLASS  *(Dmnl)* | Constant |  | 1 |  | -Tingsanchali and Promping, 2022  -https://www.postcodearea.co.uk/postaltowns/london/se280hs/demographics/ |
| WEIGHT OF FLOOD ADAPTIVE CAPACITY COMPONENTS  *(Dmnl)* | Constant |  | 0.15 |  |  |
| flood adaptive capacity factor  *(Dmnl)* | Auxiliary | (class of preparedness towards flooding+delayed citizens' involvement class  +FLOOD MONITORING AND WARNING SYSTEMS EFFECTIVENESS CLASS+HOUSEHOLDERS EDUCATION CLASS  +INFORMATION SYSTEMS EFFECTIVENESS CLASS+institutional capacity to cope with flooding over time)*WEIGHT OF FLOOD ADAPTIVE CAPACITY COMPONENTS+(DRAINAGE SYSTEMS CHARACTERISTICS CLASS*DRAINAGE SYSTEMS CHARACTERISTICS WEIGHT) |  |  | -Kissi et al. 2015  -Ntajal et al. 2016  -Nasiri et al. 2017  -Babanawo et al. 2022  -Hamidi et al. 2022  -Tingsanchali and Promping, 2022 |
|  |  | **FLOOD VULNERABILITY SUB-MODEL** |  |  |  |
| EXPOSURE FACTOR WEIGHT  *(Dmnl)* | Constant |  | 0.63 |  | -Tingsanchali and Promping, 2022 |
| SUSCEPTIBILITY FACTOR WEIGHT  *(Dmnl)* | Constant |  | 0.26 |  | -Tingsanchali and Promping, 2022 |
| ADAPTIVE CAPACITY FACTOR WEIGHT  *(Dmnl)* | Constant |  | 0.11 |  | -Tingsanchali and Promping, 2022 |
| flood vulnerability index  *(Dmnl)* | Auxiliary | EXPOSURE FACTOR WEIGHT*flood exposure factor+SUSCEPTIBILITY FACTOR WEIGHT*flood susceptibility factor-ADAPTIVE CAPACITY FACTOR WEIGHT  *flood adaptive capacity factor |  |  | -Kissi et al. 2015  -Ntajal et al. 2016  -Nasiri et al. 2017  -Babanawo et al. 2022  -Hamidi et al. 2022  -Tingsanchali and Promping, 2022 |
|  |  | **FLOOD RISK ASSESSMENT SUB-MODEL** |  |  |  |
| flood risk index  *(Dmnl)* | Auxiliary | flood hazard index*flood vulnerability index |  |  | -Kissi et al. 2015  -Ntajal et al. 2016  -Nasiri et al. 2017  -Babanawo et al. 2022  -Hamidi et al. 2022  -Tingsanchali and Promping, 2022 |
| global flood risk index  *(Dmnl)* | Auxiliary with Lookup | flood risk index    ([(0,0)-(25,10)], (1,1),(5,2),(10,2),(15,2),(20,3),(25,3) ) |  |  |  |
|  |  | **URBAN FLOOD RESILIENCE SECTION** |  |  | · |
| effect of residents' well-being on urban flood resilience  *(Dmnl)* | Auxiliary with Lookup | Residents’ Well-Being    ([(0,0)-(10,10)],(0,1),  (0.5,2),(1,3) ) |  |  | -Cutter et al. 2008  -Cutter et al. 2010  -Verrucci et al. 2012  -Batica et al. 2013  -Cutter et al. 2014  -Joerin et al. 2014  -Rockefeller, 2015  -Figureido et al. 2018  -Moghadas et al. 2019  -Feofilovs et al. 2020  -Satour et al. 2021  -Marasco et al. 2022 |
| population characteristics  *(Dmnl)* | Auxiliary with Lookup | ELDERLY AND CHILDREN CLASS    ([(0,0)-(10,10)],(1,3),  (2,2),(3,1) ) |  |  | -Cutter et al. 2008  -Cutter et al. 2010  -Verrucci et al. 2012  -Batica et al. 2013  -Cutter et al. 2014  -Joerin et al. 2014  -Rockefeller, 2015  -Figureido et al. 2018  -Moghadas et al. 2019  -Feofilovs et al. 2020  -Satour et al. 2021  -Marasco et al. 2022 |
| effect of risk perception on urban resilience  *(Dmnl)* | Auxiliary with Lookup | Community Flood Risk Perception    ([(0,0)-(10,10)],(0,1),  (0.5,2),(1,3) ) |  |  | -Cutter et al. 2008  -Cutter et al. 2010  -Verrucci et al. 2012  -Batica et al. 2013  -Cutter et al. 2014  -Joerin et al. 2014  -Rockefeller, 2015  -Figureido et al. 2018  -Moghadas et al. 2019  -Feofilovs et al. 2020  -Satour et al. 2021  -Marasco et al. 2022 |
| effect of imperviousness on urban flood resilience  *(Dmnl)* | Auxiliary with Lookup | imperviousness coefficient    ([(0,0)-(10,10)],(0,3),  (0.5,2),(1,1) ) |  |  | -Cutter et al. 2008  -Cutter et al. 2010  -Verrucci et al. 2012  -Batica et al. 2013  -Cutter et al. 2014  -Joerin et al. 2014  -Rockefeller, 2015  -Figureido et al. 2018  -Moghadas et al. 2019  -Feofilovs et al. 2020  -Satour et al. 2021  -Marasco et al. 2022 |
| effect of attractiveness on urban flood resilience  *(Dmnl)* | Auxiliary with Lookup | Attractiveness for Companies    ([(0,0)-(10,10)],(0,1),(0.5,2),(1,3) ) |  |  | -Cutter et al. 2008  -Cutter et al. 2010  -Verrucci et al. 2012  -Batica et al. 2013  -Cutter et al. 2014  -Joerin et al. 2014  -Rockefeller, 2015  -Figureido et al. 2018  -Moghadas et al. 2019  -Feofilovs et al. 2020  -Satour et al. 2021  -Marasco et al. 2022 |
| effect of ecosystem quality state on urban flood resilience  *(Dmnl)* | Auxiliary with Lookup | Ecosystem Quality State    ([(0,0)-(10,10)],(0,1),(0.5,2),(1,3) ) |  |  | -Cutter et al. 2008  -Cutter et al. 2010  -Verrucci et al. 2012  -Batica et al. 2013  -Cutter et al. 2014  -Joerin et al. 2014  -Rockefeller, 2015  -Figureido et al. 2018  -Moghadas et al. 2019  -Feofilovs et al. 2020  -Satour et al. 2021  -Marasco et al. 2022 |
| flood mitigation infrastructure effectiveness  *(Dmnl)* | Auxiliary with Lookup | flood hazard index    ([(0,0)-(10,10)],(1,3),  (2,2),(3,1) ) |  |  | -Cutter et al. 2008  -Cutter et al. 2010  -Verrucci et al. 2012  -Batica et al. 2013  -Cutter et al. 2014  -Joerin et al. 2014  -Rockefeller, 2015  -Figureido et al. 2018  -Moghadas et al. 2019  -Feofilovs et al. 2020  -Satour et al. 2021  -Marasco et al. 2022 |
| urban performance index  *(Dmnl)* | Auxiliary | (built environment quality class+ CRITICAL INFRASTRUCTURE PRESENCE CLASS+ effect of attractiveness on urban flood resilience+  effect of ecosystem quality state on urban flood resilience  + population characteristics+ effect of imperviousness on urban flood resilience+ effect of residents' well-being on urban flood resilience  +effect of risk perception on urban resilience+ institutional capacity to cope with flooding over time+ flood mitigation infrastructure effectiveness) *WEIGHT OF URBAN FLOOD RESILIENCE COMPONENTS |  |  | -Cutter et al. 2008  -Cutter et al. 2010  -Verrucci et al. 2012  -Batica et al. 2013  -Cutter et al. 2014  -Joerin et al. 2014  -Rockefeller, 2015  -Figureido et al. 2018  -Moghadas et al. 2019  -Feofilovs et al. 2020  -Satour et al. 2021  -Marasco et al. 2022 |

**Supplementary Material Section 3.** Changed variables in the modelled scenarios.

| **VARIABLE** | **BASELINE SCENARIO** | **SCENARIO 1** | **SCENARIO 2** | **SCENARIO 3** |
| --- | --- | --- | --- | --- |
| Stormwater system  capacity  *(mm/Day)* | [(0,0)-(22266,200)],(1,5),(3,104),(179,4.9),(181,103),(729,4.8),(731,97),(879,4.7),(881,95),(1094,4.6),(1096,94),(1244,4.5),(1246,92),(1460,4.4),(1462,90),(1609,4.3),(1701,89),(1824,4.2),(1826,86),(1974,4.1),(1976,85),(2189,4),(2191,83),(2339,3.9),(2341,81),(2554,3.8),(2556,79),(2704,3.7),(2706,78),(2919,3.6),(2921,76),(3069,3.5),(3071,74),(3284,3.4),(3286,72),(3434,3.3),(3436,71),(3649,3.2),(3651,68),(3799,3.1),(3801,67),(4014,3),(4016,65),(4164,2.9),(4166,63),(4379,2.8),(4381,61),(4529,2.7),(4531,60),(4744,2.6),(4746,58),(4894,2.5),(4896,56),(5110,2.4),(5112,54),(5259,2.3),(5261,53),(5474,2.1),(5476,50),(5839,2),(5841,47),(5989,1.9),(5991,45),(6204,1.8),(6206,43),(6354,1.7),(6356,41),(6569,1.6),(6571,39),(6719,1.5),(6721,37),(6935,1.4),(6937,35),(7084,1.3),(7086,33),(7299,1.2),(7301,31),(7449,1.1),(7451,29),(7664,1),(7666,27),(7814,0.9),(7816,25),(8029,0.8),(8031,23),(8179,0.7),(8181,21),(8394,0.6),(8396,19),(8544,0.5),(8546,17),(8760,0),(8762,15),(8909,0),(8911,13),(9124,0),(9126,11),(9274,0),(9276,9),(9489,0),(9491,7),(9639,0),(9641,5),(9854,0),(9856,3),(10004,0),(10006,1),(10219,0),(10221,0),(10950,0),(12775,0),(14600,0),(16425,0),(18250,0),(20075,0),(21900,0),(23725,0),(25550,0),(27375,0) | [(0,0)-(22266,200)],(1,5),(3,104),(179,4.9),(181,103),(729,4.8),(731,97),(879,4.7),(881,95),(1094,4.6),(1096,94),(1244,4.5),(1246,92),(1460,4.4),(1462,90),(1609,4.3),(1701,89),(1824,4.2),(1826,86),(1974,4.1),(1976,85),(2189,4),(2191,83),(2339,3.9),(2341,81),(2554,3.8),(2556,79),(2704,3.7),(2706,78),(2919,3.6),(2921,76),(3069,3.5),(3071,74),(3284,3.4),(3286,72),(3434,3.3),(3436,71),(3649,3.2),(3651,68),(3799,3.1),(3801,67),(4014,3),(4016,65),(4164,2.9),(4166,63),(4379,2.8),(4381,61),(4529,2.7),(4531,60),(4744,2.6),(4746,58),(4894,2.5),(4896,56),(5110,2.4),(5112,54),(5259,2.3),(5261,53),(5474,2.2),(5476,50),(5839,2.1),(5841,47),(5989,2),(5991,45),(6204,1.9),(6206,43),(6354,1.8),(6356,41),(6569,1.7),(6571,39),(6719,1.6),(6721,37),(6935,1.5),(6937,35),(7084,1.4),(7086,33),(7299,1.3),(7301,31),(7449,1.2),(7451,29),(7664,1.1),(7666,27),(7814,1),(7816,25),(8029,0.9),(8031,23),(8179,0.8),(8181,21),(8394,0.7),(8396,19),(8544,0.6),(8546,17),(8760,144),(10031,6.5),(10033,131),(10259,6.4),(10261,129),(10585,126),(10899,6.3),(10901,123),(11122,6.2),(11124,121),(11300,6.1),(11302,119),(11519,6),(11521,117),(11657,5.9),(11659,115),(11683,5.8),(11685,115),(11694,5.7),(11696,115),(11698,5.6),(11700,115),(11736,5.5),(11738,115),(12045,5.4),(12047,112),(12225,5.3),(12227,110),(12410,5.2),(12412,108),(12590,5.1),(12592,106),(12775,5),(12777,104),(12955,4.9),(12957,103),(13505,4.8),(13507,97),(13655,4.7),(13657,95),(13870,4.6),(13872,94),(14020,4.5),(14022,92),(14235,4.4),(14237,90),(14385,4.3),(14387,89),(14600,4.2),(14602,86),(14750,4.1),(14752,85),(14965,4),(14967,83),(15115,3.9),(15117,81),(15330,3.8),(15332,79),(15480,3.7),(15482,78),(15695,3.6),(15697,76),(15845,3.5),(15847,74),(16060,3.4),(16062,72),(16210,3.3),(16212,71),(16425,3.2),(16427,68),(16575,3.1),(16577,67),(16790,3),(16792,65),(16940,2.9),(16942,63),(17155,2.8),(17157,61),(17305,2.7),(17307,60),(17520,2.6),(17522,58),(17670,2.5),(17672,56),(17885,2.4),(17887,54),(18035,2.3),(18037,53),(18250,2.1),(18252,50),(18615,2),(18617,47),(18765,1.9),(18767,45),(18980,1.8),(18982,43),(19130,1.7),(19132,41),(19345,1.6),(19347,39),(19495,1.5),(19497,37),(19710,1.4),(19712,35),(19860,1.3),(19862,33),(20075,1.2),(20077,31),(20225,1.1),(20227,29),(20440,1),(20442,27),(20590,0.9),(20592,25),(20805,0.8),(20807,23),(20955,0.7),(20957,21),(21170,0.6),(21172,19),(21320,0.5),(21322,17),(21900,144),(23171,6.5),(23173,131),(23399,6.4),(23401,129),(23725,126),(24039,6.3),(24041,123),(24262,6.2),(24264,121),(24440,6.1),(24442,119),(24659,6),(24661,117),(24987,5.9),(24989,115),(24823,5.8),(24825,115),(24834,5.7),(24836,115),(24838,5.6),(24840,115),(24876,5.5),(24877,115),(25185,5.4),(25187,112),(25365,5.3),(25367,110),(25550,5.2),(25552,108),(25730,5.1),(25732,106),(25915,5),(25917,104),(26095,4.9),(26097,103),(26645,4.8),(26647,97),(26795,4.7),(26797,95),(27010,4.6),(27012,94),(27160,4.5),(27162,92),(27375,4.4) | [(0,0)-(22266,200)],(1,5),(3,104),(179,4.9),(181,103),(729,4.8),(731,97),(879,4.7),(881,95),(1094,4.6),(1096,94),(1244,4.5),(1246,92),(1460,4.4),(1462,90),(1609,4.3),(1701,89),(1824,4.2),(1826,86),(1974,4.1),(1976,85),(2189,4),(2191,83),(2339,3.9),(2341,81),(2554,3.8),(2556,79),(2704,3.7),(2706,78),(2919,3.6),(2921,76),(3069,3.5),(3071,74),(3284,3.4),(3286,72),(5110,62),(6935,52),(8760,42),(10950,32),(12775,22),(14600,12),(16425,0),(18250,0),(20075,0),(21900,0),(23725,0),(25550,0),(27375,0) | [(0,0)-(22266,200)],(1,5),(3,104),(179,4.9),(181,103),(729,4.8),(731,97),(879,4.7),(881,95),(1094,4.6),(1096,94),(1244,4.5),(1246,92),(1460,4.4),(1462,90),(1609,4.3),(1701,89),(1824,4.2),(1826,86),(1974,4.1),(1976,85),(2189,4),(2191,83),(2339,3.9),(2341,81),(2554,3.8),(2556,79),(2704,3.7),(2706,78),(2919,3.6),(2921,76),(3069,3.5),(3071,74),(3284,3.4),(3286,65),(5110,56),(6935,48),(8760,38),(10950,29),(12775,20),(14600,11),(16425,0),(18250,0),(20075,0),(21900,0),(23725,0),(25550,0),(27375,0) |
| Canals  capacity  (mm/Day) | [(0,0)-(22266,20000)],(1,760),(1460,685),(3285,548),(5110,411),(6935,274),(8760,137),(10950,0),(12775,0),(14600,0),(16425,0),(18250,0),(20075,0),(21900,0),(23725,0),(25550,0),(27375,0) | [(0,0)-(22266,20000)],(1,760),(1460,685),(3285,548),(5110,411),(6935,274),(8760,137),(10950,760),(12775,760),(14600,760),(16425,685),(18250,548),(20075,411),(21900,274),(23725,137),(25550,0),(27375,760) | [(0,0)-(22266,20000)],(1,760),(1460,685),(3285,548),(5110,457),(6935,366),(8760,275),(10950,184),(12775,93),(14600,2),(16425,0),(18250,0),(20075,0),(21900,0),(23725,0),(25550,0),(27375,0) | [(0,0)-(22266,20000)],(1,760),(1460,685),(3285,541),(5110,450),(6935,359),(8760,268),(10950,177),(12775,86),(14600,0),(16425,0),(18250,0),(20075,0),(21900,0),(23725,0),(25550,0),(27375,0) |
| Pumps  capacity  *(mm/Day)* | [(0,0)-(22266,5000)],(1,4450),(1460,4005),(3285,3560),(5110,3115),(6935,2670),(8760,2225),(10950,1780),(12775,1335),(14600,890),(16425,445),(18250,0),(20075,0),(21900,0),(23725,0),(25550,0),(27375,0) | [(0,0)-(22266,5000)],(1,4450),(1460,4005),(3285,3560),(5110,3115),(6935,2670),(8760,2225),(10950,1780),(12775,1335),(14600,890),(16425,445),(18250,4450),(20075,4005),(21900,3560),(23725,3115),(25550,2670),(27375,2225) | [(0,0)-(22266,5000)],(1,4450),(1460,4005),(3285,3560),(5110,3204),(6935,2848),(8760,2492),(10950,2136),(12775,1780),(14600,1424),(16425,1068),(18250,712),(20075,356),(21900,0),(23725,0),(25550,0),(27375,0) | [(0,0)-(22266,5000)],(1,4450),(1460,4005),(3285,3553),(5110,3197),(6935,2841),(8760,2485),(10950,2129),(12775,1773),(14600,1417),(16425,1061),(18250,705),(20075,349),(21900,0),(23725,0),(25550,0),(27375,0) |
| Community sense of safety  (Dmnl) | 1 (low class) | [(0,0)-(22266,10)],(1,1),(1460,1),(3285,1),(5110,1),(6935,1),(8760,2),(10950,2),(12775,3),(14600,3),(16425,3),(18250,3),(20075,3),(21900,3),(23725,3),(25550,3),(27375,3) | [(0,0)-(22266,10)],(1,1),(1460,1),(3285,2),(5110,2),(6935,2),(8760,2),(10950,2),(12775,1),(14600,1),(16425,1),(18250,1),(20075,1),(21900,1),(23725,1),(25550,1),(27375,1) | [(0,0)-(22266,10)],(1,1),(1460,1),(3285,2),(5110,2),(6935,2),(8760,2),(10950,2),(12775,2),(14600,2),(16425,2),(18250,2),(20075,2),(21900,2),(23725,2),(25550,2),(27375,2) |
| Citizens’  involvement  (Dmnl) | 1 (low class) | 1 (low class) | 1 (low class) | [(0,0)-(22266,10)],(1,1),(1460,2),(3285,3),(5110,3),(6935,3),(8760,3),(10950,3),(12775,3),(14600,2),(16425,2),(18250,2),(20075,2),(21900,2),(23725,2),(25550,2),(27375,2) |
| Wetlands  area  *(sqkm)* | — | — | — | [(0,0)-(22266,10)],(1,0),(1460,0),(3285,0.31),(5110,0.31),(6935,0.31),(8760,0.31),(10950,0.31),(12775  ,0.31),(14600,0.31),(16425,0.31),(18250,0.31),(20075,0.31),(21900,0.31),(23725,0.31),(25550,0.31),(27375,0.31) |
| Wetlands  hydrological performance  *(mm/Day)* | — | — | — | [(0,0)-(22266,10)],(1,0),(1460,0),(3285,0.9),(5110,0.9),(6935,0.9),(8760,0.9),(10950,0.9),(12775,0.9),(14600,0.9),(16425,0.9),(18250,0.9),(20075,0.9),(21900,0.9),(23725,0.9),(25550,0.9),(27375,0.9) |
| Urban green avenue  /woodland area  *(sqkm)* | — | — | — | [(0,0)-(22266,10)],(1,0),(1460,0),(3285,0.15),(5110,0.2),(6935,0.25),(8760,0.3),(10950,0.35),(12775  ,0.36),(14600,0.36),(16425,0.36),(18250,0.36),(20075,0.36),(21900,0.36),(23725,0.36),(25550,0.36),(27375,0.36) |
| Urban green avenue /woodland hydrological performance  *(mm/Day)* | — | — | — | [(0,0)-(22266,10)],(1,0),(1460,0),(3285,0.62),(5110,0.62),(6935,0.62),(8760,0.62),(10950,0.62),(12775,0.62),(14600,0.62),(16425,0.62),(18250,0.62),(20075,0.62),(21900,0.62),(23725,0.62),(25550,0.62),(27375,0.62) |
| Intensive Blue/Green roofs area  *(sqkm)* | — | — | — | [(0,0)-(22266,10)],(1,0),(1460,0),(3285,0.02),(5110,0.025),(6935,0.03),(8760,0.035),(10950,0.04),(12775  ,0.04),(14600,0.04),(16425,0.04),(18250,0.04),(20075,0.04),(21900,0.04),(23725,0.04),(25550,0.04),(27375,0.04) |
| Intensive Blue/Green roofs hydrological performance  *(mm/Day)* | — | — | — | [(0,0)-(22266,10)],(1,0),(1460,0),(3285,0.75),(5110,0.75),(6935,0.75),(8760,0.75),(10950,0.75),(12775,0.75),(14600,0.75),(16425,0.75),(18250,0.75),(20075,0.75),(21900,0.75),(23725,0.75),(25550,0.75),(27375,0.75) |
| Parks area  *(sqkm)* | — | — | — | [(0,0)-(22266,10)],(1,0),(1460,0),(3285,0.62),(5110,0.62),(6935,0.62),(8760,0.62),(10950,0.62),(12775  ,0.62),(14600,0.62),(16425,0.62),(18250,0.62),(20075,0.62),(21900,0.62),(23725,0.62),(25550,0.62),(27375,0.62) |
| Parks  hydrological performance  *(mm/Day)* | — | — | — | [(0,0)-(22266,10)],(1,0),(1460,0),(3285,1),(5110,1),(6935,1),(8760,1),(10950,1),(12775,1),(14600,1),(16425,1),(18250,1),(20075,1),(21900,1),(23725,1),(25550,1),(27375,1) |
| Lakes and canals  naturalization  *(Dmnl)* | — | — | — | [(0,0)-(27375,10)],(1,0),(1460,0),(3285,0.05),(5110,0.05),(6935,0.05),(8760,0.05),(10950,0.05),(12775  ,0.05),(14600,0.05),(16425,0.05),(18250,0.05),(20075,0.05),(21900,0.05),(23725,0.05),(25550,0.05),(27375,0.05) |
| Proximity to natural spaces  *(Dmnl)* | 0.5 (medium level) | 0.5 (medium level) | 0.5 (medium level) | [(0,0)-(22266,10)],(1,0.5),(1460,0.5),(3285,0.52),(5110,0.54),(6935,0.56),(8760,0.58),(10950,0.6),(12775  ,0.62),(14600,0.64),(16425,0.66),(18250,0.68),(20075,0.7),(21900,0.72),(23725,0.74),(25550,0.76),(27375,0.78) |
| Wetlands  biodiversity performance  *(Dmnl/Day)* |  |  |  | [(0,0)-(22266,10)],(1,0),(1460,0),(3285,1.5e06),(5110,1.5e06), (6935,1.5e-06),(8760,1.5e-06),(10950,1.5e-06), (12775,1.5e-06), (14600,1.5e-06), (16425,1.5e-06), (18250,1.5e-06), (20075,1.5e-06), (21900,1.5e-06), (23725,1.5e-06), (25550,1.5e-06), (27375,1.5e-06) |
| Urban green avenue /woodland biodiversity performance  *(Dmnl/Day)* | — | — | — | [(0,0)-(22266,10)],(1,0),(1460,0),(3285,1.5e06),(5110,1.5e06), (6935,1.5e-06),(8760,1.5e-06),(10950,1.5e-06), (12775,1.5e-06), (14600,1.5e-06), (16425,1.5e-06), (18250,1.5e-06), (20075,1.5e-06), (21900,1.5e-06), (23725,1.5e-06), (25550,1.5e-06), (27375,1.5e-06) |
| Intensive Blue/Green roofs  biodiversity performance  *(Dmnl/Day)* | — | — | — | [(0,0)-(22266,10)],(1,0),(1460,0),(3285,1.5e06),(5110,1.5e06), (6935,1.5e-06),(8760,1.5e-06),(10950,1.5e-06), (12775,1.5e-06), (14600,1.5e-06), (16425,1.5e-06), (18250,1.5e-06), (20075,1.5e-06), (21900,1.5e-06), (23725,1.5e-06), (25550,1.5e-06), (27375,1.5e-06) |
| Parks  biodiversity performance  *(Dmnl/Day)* | — | — | — | [(0,0)-(22266,10)],(1,0),(1460,0),(3285,1.5e06),(5110,1.5e06), (6935,1.5e-06),(8760,1.5e-06),(10950,1.5e-06), (12775,1.5e-06), (14600,1.5e-06), (16425,1.5e-06), (18250,1.5e-06), (20075,1.5e-06), (21900,1.5e-06), (23725,1.5e-06), (25550,1.5e-06), (27375,1.5e-06) |
